# Metabolic and stress responses of *Acinetobacter oleivorans* DR1 during long‐chain alkane degradation

**DOI:** 10.1111/1751-7915.12852

**Published:** 2017-08-31

**Authors:** Chulwoo Park, Bora Shin, Jaejoon Jung, Yunho Lee, Woojun Park

**Affiliations:** ^1^ Laboratory of Molecular Environmental Microbiology Department of Environmental Science and Ecological Engineering Korea University Seoul 02841 Korea; ^2^ National Marine Biodiversity Institute of Korea Chungcheongnam‐Do 33662 Korea; ^3^ Department of Life Science Chung‐Ang University Seoul 06974 Korea

## Abstract

*Acinetobacter oleivorans* DR1 can utilize C_12_–C_30_ alkanes as a sole carbon source but not short‐chain alkanes (C_6_, C_10_). Two copies of each *alkB*‐, *almA*‐ and *ladA*‐type alkane hydroxylase (AH) are present in the genome of DR1 cells. Expression and mutational analyses of AHs showed that *alkB1* and *alkB2* are the major AH‐encoding genes under C_12_–C_30_, and the roles of other *almA*‐ and *ladA* genes are negligible. Our data suggested that AlkB1 is responsible for long‐chain alkane utilization (C_24_–C_26_), and AlkB2 is important for medium‐chain alkane (C_12_–C_16_) metabolism. Phylogenetic analyses revealed large incongruities between phylogenies of 16S rRNA and each AH gene, which implies that *A. oleivorans* DR1 has acquired multiple alkane hydroxylases through horizontal gene transfer. Transcriptomic and qRT‐PCR analyses suggested that genes participating in the synthesis of siderophore, trehalose and poly 3‐hydroxybutyrate (PHB) were expressed at much higher levels when cells used C_30_ than when used succinate as a carbon source. The following biochemical assays supported our gene expression analyses: (i) quantification of siderophore, (ii) measurement of trehalose and (iii) observation of PHB storage. Interestingly, highly induced both *ackA* gene encoding an acetate kinase A and *pta* gene encoding a phosphotransacetylase suggested unusual ATP synthesis during C_30_ alkane degradation, which was demonstrated by ATP measurement using the *ΔackA* mutant. Impaired growth of the *ΔaceA* mutant indicated that the glyoxylate shunt pathway is important when C_30_ alkane is utilized. Our data provide insight into long‐chain alkane degradation in soil microorganisms.

## Introduction

A large number of hydrocarbonoclastic‐ and alkane‐degrading bacteria are widely distributed in nature (Liu *et al*., 2015). Their degrading mechanisms have been investigated owing to their ecological importance and the versatile applications of alkane‐degrading enzymes with economic benefits (Rojo, 2010). Four different pathways for aerobic alkane oxidation have been reported: terminal oxidation, subterminal oxidation, biterminal oxidation and the Finnerty pathway (Ji *et al*., 2013). The best‐characterized strategy for aerobic alkane degradation is terminal oxidation wherein through a series of oxidation steps, the alkane is transformed into alcohol, aldehyde and, finally, into fatty acid (Van Beilen *et al*., 2003). The first reaction mediated by alkane hydroxylase is known to be the rate‐limiting step, which converts alkane to fatty alcohol. Diverse alkane hydroxylases, from many different bacteria, have been characterized, revealing that they have different ranges of carbon‐chain length preferences with respect to alkane substrates (Van Beilen and Funhoff, 2007). Short‐chain alkanes (C_1_–C_4_) can be oxidized by soluble methane monooxygenase, particulate methane monooxygenase, propane monooxygenase and butane monooxygenase while integral‐membrane non‐haem di‐iron monooxygenase (AlkB or AlkM) and alkane hydroxylating cytochrome P450 appear to mediate the oxidation of short‐ and medium‐chain alkanes (C_5_–C_16_). In addition, the recently discovered novel monooxygenases, such as AlmA in *Acinetobacter* sp. DSM 17978 and LadA in *Geobacillus thermodenitrificans* NG80‐2, have gained attention because they can oxidize very long‐chain alkanes (> C_20_), indicating that these enzymes could potentially be used for bioremediation because of their unusual substrate ranges (Feng *et al*., 2007; Throne‐Holst *et al*., 2007).

Examination of novel alkane dioxygenase in *Acinetobacter* sp. M‐1 has revealed activity on the C_10_–C_30_ alkanes through postulated Finnerty pathway (Maeng *et al*., 1996). Genomic data do not reveal the substrate ranges of each alkane hydroxylase, and the regulation of gene expression involved in alkane degradation is not well understood in many known alkane degraders. Expression analysis of the alkane hydroxylase genes provides insight into how bacteria can utilize alkanes and which enzymes should be investigated for biotechnological applications. Expression of the *alkM* gene in *Acinetobacter* sp. ADP1 is variable depending on its carbon source, growth phase and inducer molecules, although rubredoxin and rubredoxin reductase, which are the components of the full alkane hydroxylase complex, are constitutively expressed (Ratajczak *et al*., 1998). In addition, *Alcanivorax dieselolei* B‐5, which contains at least four alkane hydroxylases, degrades a wide range of alkanes (C_5_–C_36_); these multiple alkane hydroxylases are coexpressed and were shown to degrade a broad range of alkanes with different chain length alkanes (Liu *et al*., 2011). *Acinetobacter* species have been extensively studied because of their versatile ability to degrade diverse hydrocarbons and their potential biotechnological applications (Jung and Park, 2015). It has been determined that many *Acinetobacte*r strains have the ability to utilize long‐chain alkanes (> C_20_) as a carbon source (Wentzel *et al*., 2007). In addition, *Acinetobacter calcoaceticus* uses hydrophobic fimbriae, which assists in attachment to the hydrophobic surface of substrates (Rosenberg *et al*., 1982), and *Acinetobacter venetianus* RAG‐1 produces a kind of polysaccharide biosurfactants, known as an emulsan, which facilitates incorporation of alkanes into bacterial cells (Bach *et al*., 2003). However, the mechanisms of alkane transport and the enzymes involved in alkane degradation in other *Acinetobacter* species, including *Acinetobacter oleivorans* DR1, are not well understood (Kang and Park, 2009).

In this study, we determined the range of alkanes that strain DR1 can use as a carbon source. Expression patterns of putative alkane monooxygenases on a medium‐chain alkane (C_12_) to a long‐chain alkane (C_30_) were also monitored using quantitative reverse transcription polymerase chain reaction (qRT‐PCR) and Northern blot. In addition, the role of each gene was confirmed by gene deletion analysis. Phylogenetic analysis demonstrated that strain DR1 possesses six putative alkane monooxygenases. Finally, RNA‐seq analysis of strain DR1 in the presence of triacontane (C_30_) was conducted. Our analyses also provide considerable insight into metabolic and stress responses during bacterial long‐chain alkane metabolism.

## Results

### Substrate ranges of alkane degradation in *A. oleivorans* DR1

**Figure 1:**
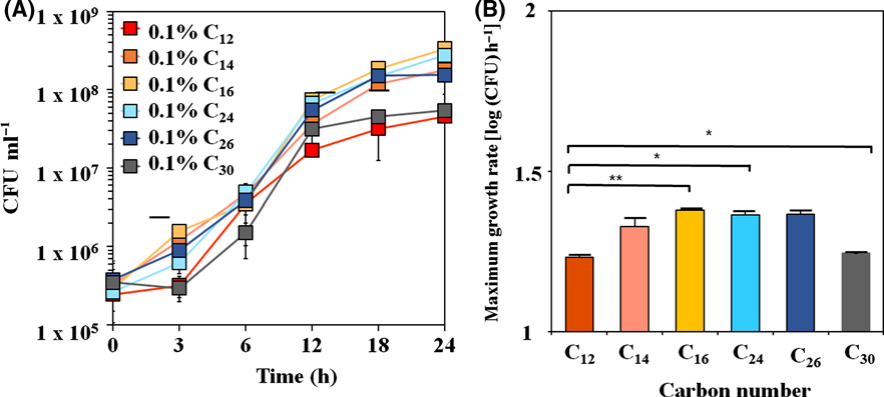
Determination of substrate range of the DR1 strain was carried out using growth curves on (A) medium‐chain length alkanes (C_12_–C_16_) to long‐chain length alkanes (C_24_–C_30_). (B) Comparison of maximum growth rate based on growth curves. Duplicate experiments were performed, and each dot indicates the average of the experiments. Error bars indicate standard deviation (SD). A *t*‐test was performed, and a *P*‐value less than 0.01 or 0.05 is marked by a double or single asterisk (**P < 0.01, *P < 0.05).

The DR1 cells are known to utilize diesel and hexadecane as carbon sources (Jung *et al*., 2011a; Kang *et al*., 2011); however, the range of degradable aliphatic alkanes is not known and the corresponding alkane monooxygenases have not been characterized. Growth tests on various substrates indicated that DR1 cells could utilize both medium‐ and long‐chain alkanes (C_12_–C_30_) but not short‐chain alkanes (C_6_–C_10_) (Fig. 1A, Figs S1A and S1B). Because of the extremely low water solubility of tested alkanes (less than 0.01 mg L^−1^ at ambient temperature), the maximum growth rate could be monitored through simple growth measurements using each alkane at 0.1% concentration. The highest growth rate was observed with hexadecane (C_16_, μ_max_ = 1.38 h^−1^) followed by hexacosane (C_24_, μ_max_ = 1.37 h^−1^), tetracosane (C_26_, μ_max_ = 1.37 h^−1^) and tetradecane (C_14_, μ_max_ = 1.33 h^−1^). The lowest growth rates were determined with dodecane (C_12_, μ_max_ = 1.23 h^−1^) and triacontane (C_30_, μ_max_ = 1.25 h^−1^), which had the shortest and longest carbon‐chain lengths among tested alkanes respectively (Fig. 1B). Although no growth was detected in case of hexane, its corresponding oxidized products (hexanol and hexanoic acid) could be utilized as carbon sources (Fig. S1A). In contrast, growth occurred only with decanoic acid as carbon source but not with decanol (Fig. S1B). In addition, further examination of decanol by survival and agar diffusion tests showed its high toxicity to DR1 strains. Only 0.1% of total cells survived 15‐min postdecanol treatment (Fig. S1D). Agar diffusion tests with decane and decanol confirmed the results of the survival test. A clear zone was not formed even when 100% decane was used; however, inhibition occurred following treatment with 50% and 100% decanol, showing 2.3‐ and 2.5‐cm clear zones respectively (Fig. S1E). A high concentration of decanol (> 50%) was required to suppress the growth of DR1 in the agar diffusion test, although only 0.1% decanol was sufficient to inhibit growth in the liquid survival test. This difference may be due to the limited solubility of decanol, which hindered its diffusion on the agar disc. Our data indicated that alkane monooxygenases in DR1 cells are specific for medium‐ and long‐chain alkane degradation.

### Expression analysis and knockout mutant studies of AlkB‐type alkane monooxygenases in the presence of triacontane

Sequence analysis has revealed the presence of homologs of alkane monooxygenases (*alkB*‐, *almA*‐ and *ladA* types) in strain DR1 (Jung *et al*., 2010). Based on amino acid alignments, two copies of three different types of alkane monooxygenases were identified (Table 1). Both *alkB1* and *alkB2* homologs possessed relatively high amino acid identities with *alkM*a (88%) and *alkM*b (93%) of *Acinetobacter* sp. M‐1, respectively. AlmA and LadA homologs have high identities with those of *Acinetobacter* sp. DSM 17874 and of *Geobacillus denitrificans* NG80‐2, respectively (Table 1). However, the DR1 genome does not seem to harbour any cytochrome P450‐type monooxygenase gene.

**Table 1:** The result of alignment for alkane monooxygenase homologues between *Acinetobacter oleivorans* DR1 and other bacteria using blastp

Gene seq identifier	Gene name	Protein/organism	Amino acid identity (%)	Length of amino acids	*E*‐value
AOLE_RS10590	*alkB1*	Alkane hydroxylase A (AlkMa)/*Acinetobacter* sp. M‐1	359/409 (88%)	407	0
AOLE_RS13400	*alkB2*	Alkane hydroxylase B (AlkMb)/*Acinetobacter* sp. M‐1	368/395 (93%)	397	0
AOLE_RS02255	*almA1*	Putative monooxygenase (AlmA*)*/*Acinetobacter* sp. DSM 17874	406/497 (82%)	496	0
AOLE_RS09555	*almA2*		228/481 (47%)	510	2e−159
AOLE_RS11290	*ladA1*	Monooxygenase (LadA)/*Geobacillus thermodenitrificans* NG80‐2	211/447 (47%)	470	2e−154
AOLE_RS11525	*ladA2*		231/455 (51%)	463	7e−168

**Figure 2:**
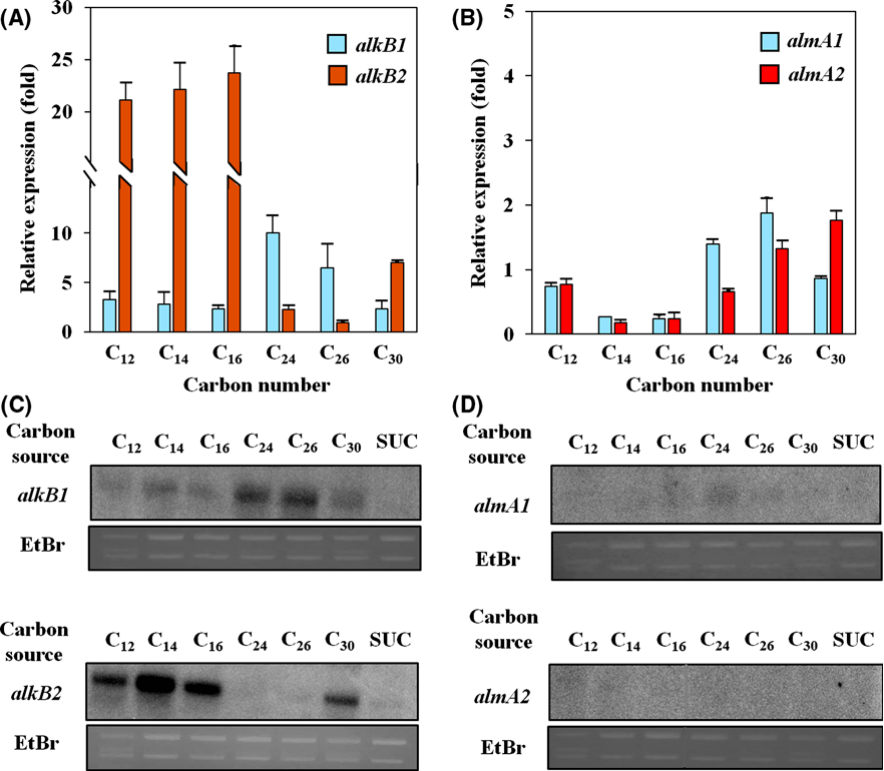
Relative expression levels of (A) *alkB1* (cyan bar), *alkB2* (red bar), (B) *almA1* (cyan bar) and *almA2* (red bar) along medium‐chain alkanes to long‐chain alkanes (C_12_, C_14_, C_16_, C_24_ and C_30_) were measured by qRT‐PCR. The fold changes are defined as the expressions of each gene with alkanes comparing to 10 mm succinate. The expression levels of each gene were normalized by 16S rDNA. Northern blotting was also performed to visualize the expression of (C) *alkB* and (D) *almA*. A consistent amount of total RNA was loaded, which is shown by the ethidium bromide‐stained gel (EtBr).

**Figure 3:**
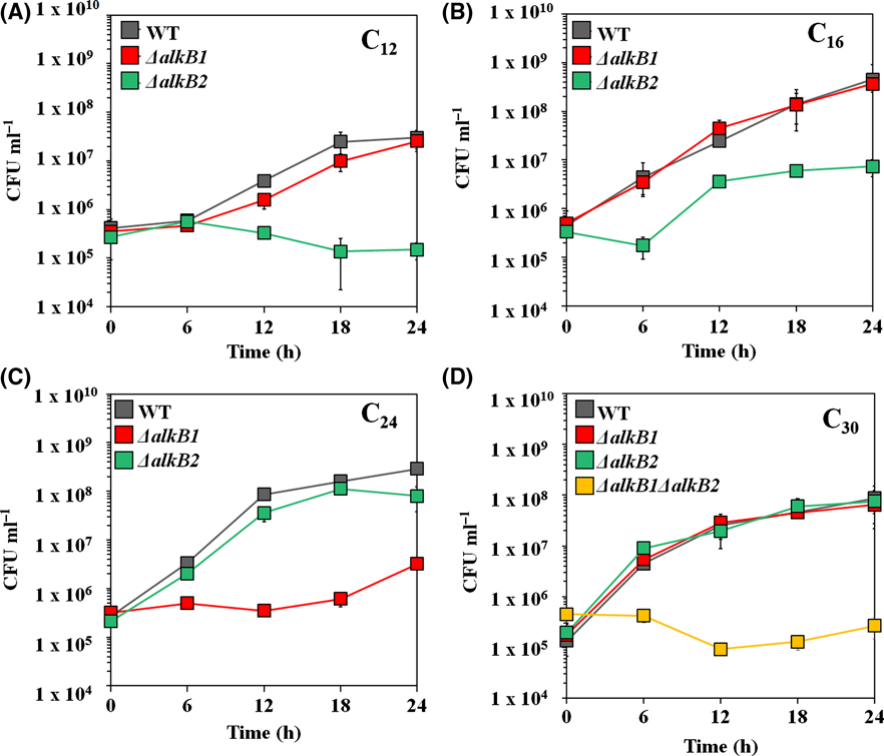
Comparative growth assay of wild type, *alkB* single‐ (*ΔalkB1* and *ΔalkB2*) and double‐knockout mutants (*ΔalkB1Δalk*B2) on (A) C_12_, (B) C_16_, (C) C_24_ and (D) C_30_ alkanes supplied with MSB media.

Transcriptomic analysis of the strain DR1 cells grown on triacontane (hereinafter TRI) was conducted, and cells grown on 10 mm succinate (hereinafter SUC) were used as controls. The information obtained from the raw RNA‐seq profile is summarized in Table S1. All genes associated with the predicted alkane degradation pathway were upregulated. However, six alkane monooxygenase‐encoding genes were differentially expressed (Table S2). *alkB1* and *alkB2* were highly expressed (11.6‐fold and 7.2‐fold, respectively) with high RPKM values, indicating that AlkB‐type monooxygenases are important for TRI degradation in DR1 cells, although other AlmA‐ and LadA‐type enzymes are known to be involved in long‐chain alkane degradation in other bacterial species (Feng *et al*., 2007; Throne‐Holst *et al*., 2007). Induction of AlkB‐type enzymes for TRI degradation was also confirmed by qRT‐PCR and Northern blot analysis (Fig. 2). The *alkB1* gene is more inducible in presence of C_24_–C_26_ alkanes, while *alk*B2 is highly expressed with C_12_–C_16_ (Fig. 2A and C). In addition, a growth defect of the *alkB2* mutant grown on C_12_ and C_16_ alkanes (Fig. 3A and B) and that of the *alkB1* mutant on the C_24_ alkane (Fig. 3C) indicated their significant roles in degrading medium‐ and long‐chain alkanes. Consistent with our RNA‐seq data, our qRT‐PCR and Northern blot data revealed that both *alkB* genes are induced with the C_30_ alkane (Fig. 2A and C). In addition, no growth was observed in the *ΔalkB1 ΔalkB2* double‐knockout strain (Fig. 3D). Unexpected observation of this expression study implies that more complex mechanisms occur during C_30_ alkane degradation, unlike C_24_ and C_26_ alkane metabolism. Our mutational study also supports our observation showing that deletion of a single *alkB* gene does not affect the growth on the C_30_ alkane (Fig. 3D). This observation may be attributed to the compensation of each *alkB* gene deletion by the remaining *alkB* genes, which could be possible because of intrinsic slow growth and expression of both the *alkB* genes in the presence of TRI. Northern blot analyses were performed to reveal the level of *alkB2* expression in the *alkB1* mutant background under C_30_‐amended condition. Interestingly, the level of *alkB2* gene expression in the *ΔalkB1* mutant was greater than that in wild‐type cells (Fig. S2). However, our data also suggested that weak *alk*B1 transcription in the *ΔalkB2* mutant might be sufficient for full growth of the mutant on TRI even in the absence of AlkB2 activity (Fig. 3D). Collectively, our data indicated that both *alkB* gene products play significant roles in the WT strain on TRI metabolism. Although our qRT‐PCR showed the induction of the *almA* genes during C_24_, C_26_ and C_30_ alkane metabolism, Northern blot analysis indicated a very low level of expression of both the *almA* genes (Fig. 2B and D). In addition, *ladA* genes appear to be not important for degradation of TRI because of their low level of expression (Table S2). Taken together, we observed that *alk*B‐type alkane hydroxylases are important for degradation of TRI, and the contribution of both *almA* and *ladA* types to C_30_ alkane degradation is negligible.

### Phylogenetic analysis of alkane monooxygenases

**Figure 4:**
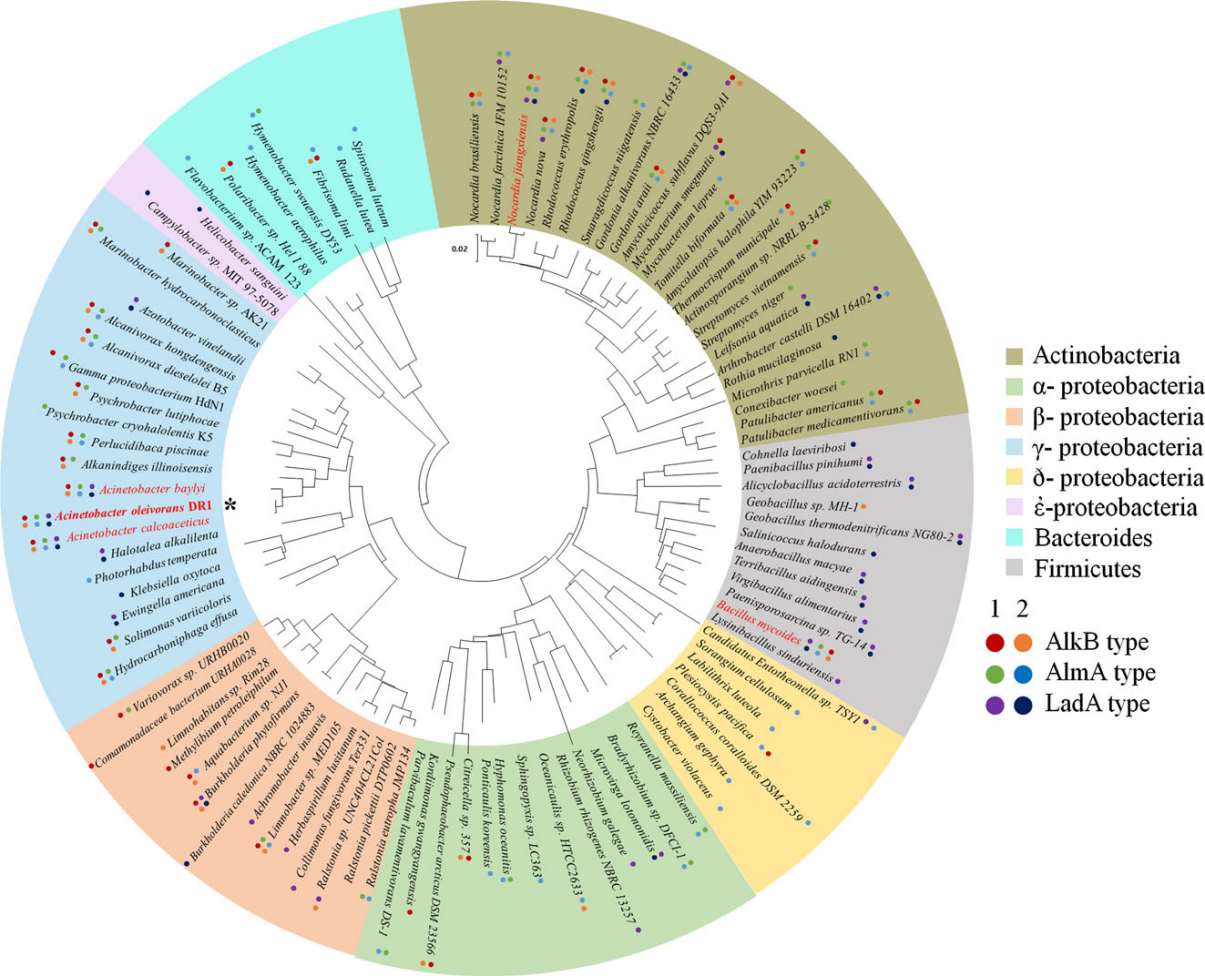
Neighbour‐joining phylogenetic tree of bacteria harbouring alkane monooxygenase homologs based on 16S rRNA. Each colour represents a phylum level of the bacterial community. The number on the right side indicates the type number of each alkane monooxygenase in the strain DR1. The scale bar represents the expected value of substitutions per point. Strain DR1 is highlighted and marked with an asterisk (*). Red letters indicate six alkane monooxygenase‐possessing bacteria.

Multiple copies of alkane monooxygenases are present in the DR1 strain possibly because of horizontal gene transfer and gene duplication events. Phylogenetic analysis showed that 13 of 19 γ‐proteobacteria species shared *alkB*‐type alkane monooxygenases (Fig. 4). In contrast, AlmA‐type and LadA‐type monooxygenases were present mostly in Actinobacteria (21 of 25 species) and Firmicutes (11 of 12 species). Although *Geobacillus* sp. MH‐1 belonging to Firmicute harbours only AlkB2 type alkane monooxygenase, LadA‐type alkane monooxygenase is more dominant in other Firmicutes, which possess only single or two copies of LadA. All three types of alkane monooxygenases found in the DR1 strain were found in only two other species, *Nocardia jianxiensis* (Actinobacteria) and *Bacillus mycoides* (Firmicutes). All examined *Acinetobacter* species (*A. oleivorans* DR1, *A. baylyi* and *A. calcoaceticus*) have six alkane monooxygenases, which indicates that the *Acinetobacter* species are specialists in degradation of alkane substrates. Results of distribution analysis of various alkane monooxygenase genes were found to be largely inconsistent with respect to results of the 16S rRNA gene phylogeny analysis; this also suggested that multiple copies of alkane monooxygenase genes occurred through horizontal gene transfer (HGT).

### Iron requirement during TRI metabolism

**Figure 5:**
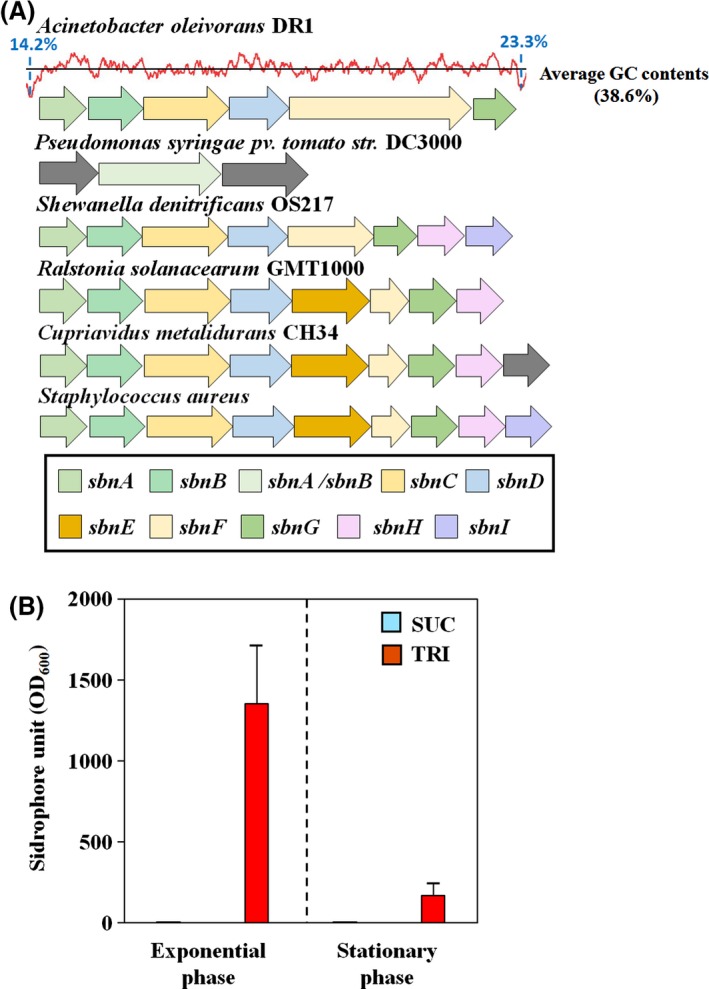
(A) AA alignment of *sbn* operon with other Gram‐negative strains and Gram‐positive *Staphylococcus aureus*. The red graph indicates GC contents in the *sbn* operon in DR1 genome, and the black line shows the average GC contents. Blue‐coloured GC contents were extremely lower than average. Different genes were coloured as shown in the boxes. (B) CAS assay for quantification of siderophore production under SUC and TRI supplementation. Siderophore production was not detected under SUC supplementation at both the exponential and stationary phase. Siderophore units were standardized by OD_600_.

The ability to scavenge iron from the environment is an important characteristic of hydrocarbon‐degrading bacteria. Iron uptake is essential for synthesis of alkane monooxygenases because non‐haem di‐iron monooxygenase (AlkB) and cytochrome P450 monooxygenase superfamily proteins (AlmA and LadA) require iron. Genes related to siderophore biosynthesis and transportation in the DR1 strain were upregulated, proving that iron requirement is high when TRI is degraded (Table S2). In the genome of the DR1 strain, two clusters associated with siderophore synthesis are present: one cluster for acinetobactin synthesis (*ent* operon, homologous to enterobactin in *Escherichia coli*) and the other cluster for staphyloferrin synthesis (*sbn* operon). Interestingly, genes ranging from AOLE_RS07230 (*sbnG*) to AOLE_RS07255 (*sbnA*) showed over 38% amino acid sequence identity with the *sbn* operon of *Staphylococcus aureus* although two *sbnE* and *sbnI* genes are missing (Fig. 5A). Based on our RNA‐seq data, the *ent* operon was not significantly induced by TRI, but the *sbn* operon was induced. This upregulation was also checked using qRT‐PCR (Fig. S3). Comparison of the *sbn* operon among a number of Gram‐negative bacteria and *Staphylococcus aureus* using blastp demonstrated high variability between the species. Further genomic analysis showed much lower GC contents upstream of *sbnA* (14.2%) and downstream of *sbnG* (23.3%) than the average (38.6%) GC content. To confirm higher production of siderophore, the CAS assay was conducted in both exponential and stationary growth phases, which showed high siderophore production in TRI‐supplemented media (Fig. 5B). Higher CAS activity was observed than control (succinate). In addition, the expression of *sbnA* was also upregulated during C_16_ degradation (Fig. S4). Collectively, the expression of *sbnA* was linked to siderophore production when DR1 cells use C_16_ as well as TRI. Three ferric siderophore receptor proteins encoding genes (*bfrZ*) were upregulated by 2.1‐ to 17.1‐fold, which is also consistent with the qRT‐PCR data (2.2‐fold change), in the media supplemented with TRI (Table S2 and Fig. S3). Our data show that highly induced *sbn* operon affected the synthesis of siderophore during TRI degradation.

### Trehalose biosynthesis during TRI degradation

**Figure 6:**
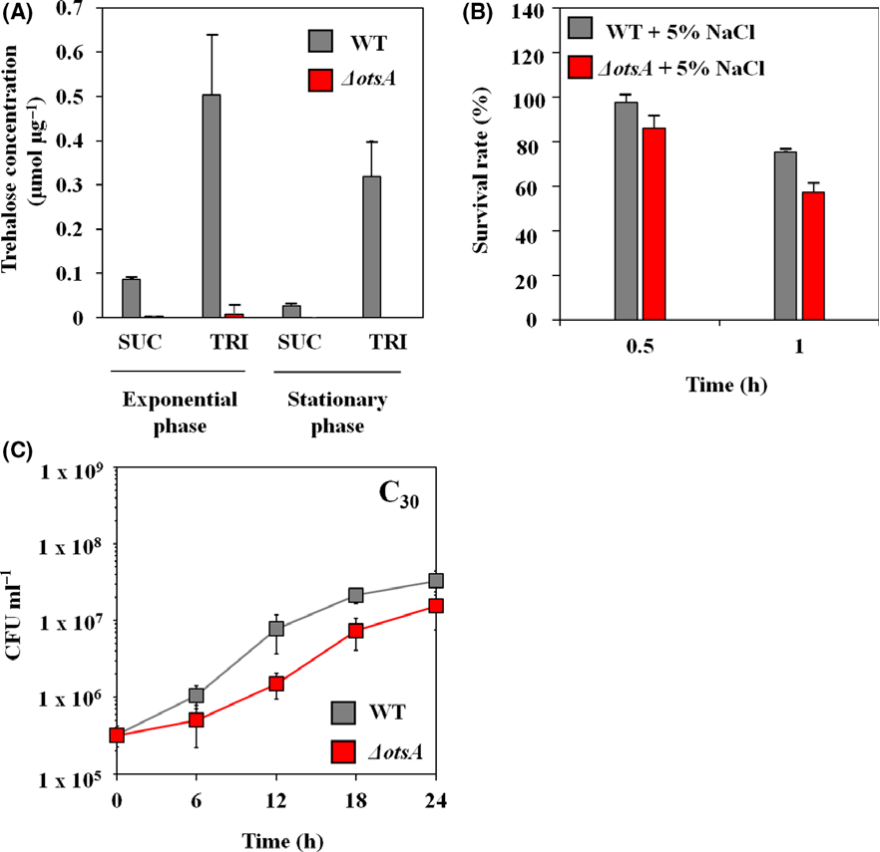
(A) Promoted trehalose production under TRI compared to SUC was confirmed using the trehalose assay. Scarcely produced trehalose was shown under SUC on the exponential and stationary phase. Trehalose was not detected on the *ΔotsA* (red bar) KO mutant even under TRI. (B) Survival rates of wild type (WT) and the *ΔotsA* mutant grown in TRI‐supplemented media were measured under PBS containing 5% NaCl for 1 h. Survival rates were calculated as follows: (CFU ml^−1^ at each time)/(CFU ml^−1^ at 0 h) × 100. (C) Growth assay of wild type (WT) and *ΔotsA* mutants in the TRI (C_30_) supplied MSB media.

The DR1 cells are known to display glucose metabolism disability due to the lack of glucokinase, which converts glucose to glucose‐6‐phosphate (Jung *et al*., 2011b). However, interestingly, trehalose (a disaccharide composed of two glucose molecules) metabolism‐related genes were highly upregulated during TRI metabolism (Table S2 and Fig. S3). In particular, the expression level of *otsA* and *otsB* genes, whose products are involved in synthesizing free trehalose from UDP‐glucose, was significantly increased (26.6‐ and 116.4‐fold respectively). Gluconeogenesis occurred during TRI degradation as indicated by high RPKM values of gluconeogenesis‐related genes (*eno*,* gpm*I, *fda*), which are associated with SUC metabolism (Table S2). Trehalose is also one of the representative alpha‐linked disaccharides synthesized intracellularly that helps cells to adapt to various stressors such as oxidative, heat, osmotic and desiccation stress (Benaroudj *et al*., 2001; Purvis *et al*., 2005; Doehlemann *et al*., 2006; Tapia *et al*., 2015). Trehalose measurement was performed using wild type and the *otsA*‐disrupted strain. Complete loss of trehalose accumulation was found only in the mutant (Fig. 6A), whereas wild‐type cells produced more trehalose in presence of TRI in both exponential and stationary growth phases. Sensitivity of the *otsA* mutant to salt stress (5% NaCl) was slightly greater than that of the wild type (Fig. 6B). In addition, growth defect of the *otsA* mutant was also observed in the presence of TRI (Fig. 6C). Collectively, these results suggest that TRI stimulates the synthesis of trehalose in the DR1 cells, which might enable cells to tolerate osmotic stress.

### Glyoxylate shunt and energy metabolism in the presence of TRI

**Figure 7:**
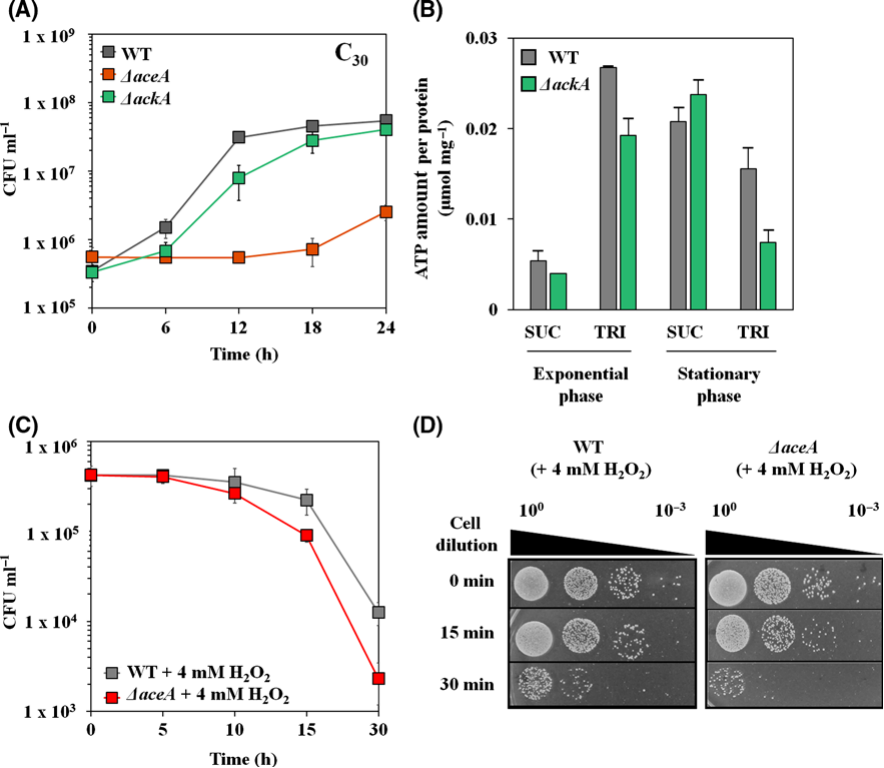
(A) Comparative growth assay of wild type (WT) and *ΔaceA*‐, *ΔackA*‐ mutants in the TRI‐supplemented media. (B) Quantification of intracellular ATP using Eliten ATP assay kit on both exponential and stationary phases. (C), (D) Comparative survival test of WT and *ΔaceA* mutant under 4 mm H_2_O_2_ (2MIC)‐treated conditions.

The tricarboxylic acid (TCA) cycle is one of the main energy generating pathways involved in production of ATP, NADPH and FADH_2_. However, most TCA cycle‐related genes were downregulated during TRI assimilation. Instead, the *aceA* (encoding an isocitrate lyase) was highly upregulated in the presence of TRI (Table S2), although the *glcB* (encoding a malate synthase G) is not induced but constitutively expressed in both conditions (Table S2). The AckA (acetate kinase A) and Pta (phosphotransacetylase) genes are involved in producing ATP during conversion of acetyl‐CoA to acetate. The DR1 genome has AckA and Pta homologs, which have 45% and 49% amino acid similarities, respectively, with those of the *E. coli* K‐12 strain. These two *ack*A and *pta* genes were upregulated by 2.8‐ and 1.3‐fold with high RPKM values (Table S2 and Fig. S3). Growth defects of *ΔaceA* and *ΔackA* mutants were observed during TRI degradation, which indicated that both the glyoxylate shunt and Ack‐Pta pathway play important roles when DR cells grow on TRI (Fig. 7A). Growth defect of the *ΔaceA* mutant was observed under acetate condition, but the mutant cells could grow with longer lag phase under either hexadecane or hexadecanoic acid metabolism (Fig. S5). Furthermore, downregulation of F0F1 ATP synthase‐encoding genes (*atp* operon) also suggested the importance of the Ack‐Pta pathway in generation of ATP when the cells metabolize TRI. The results of the ATP assay supported the results of our expression and mutant analyses (Fig. 7B). Deletion of *ackA* resulted in less ATP production during TRI degradation. The *ΔaceA* mutant showed a lower survival rate than the wild type under H_2_O_2_‐treated conditions, suggesting that the glyoxylate bypass is essential not only for TRI degradation but also under oxidative stress conditions (Fig. 7C and D). Recently, we reported the importance of the glyoxylate shunt for defending cells against oxidative stress (Ahn *et al*., 2016). Other reports revealed that the *ack*A and *pta* genes might be important under reactive oxygen species (ROS) stress (Sadykov *et al*., 2013). Taken together, the glyoxylate shunt and Ack‐Pta pathway might be essential not only for metabolizing TRI but also for protecting cells under oxidative stress, which might be a consequence of TRI degradation.

## Discussion

Since its first isolation from soil, *A. oleivorans* DR1 has been a known alkane degrader (Kang *et al*., 2011). Simple growth measurements with different lengths of alkane chains suggested that DR1 cells could grow on media containing medium‐ and long‐chain alkanes (C_12_ to C_30_) but not short‐chain alkanes (C_6_, and C_10_) (Fig. 1A and Fig. S1C). This might result from the lack of a proper alkane monooxygenase for degrading short‐chain alkanes, because DR cells could utilize short‐chain alcohols and fatty acids (Fig. S1A). However, our data also suggested that decanol toxicity occurs for *A. oleivorans* (Figs S1D and S1E), which was also reported in other microorganisms such as *Staphylococcus aureus* (Togashi *et al*., 2007) and *Mycobacteria* (Mukherjee *et al*., 2013), although the toxicity mechanism remains unclear.

Phylogenetic analysis of alkane hydroxylases showed that three *Acinetobacter* species (*A. oleivorans* DR1, *A. calcoaceticus* and *A. baylyi*) and only two non‐*Acinetobacter* bacterial strains (*Nocardia jiangxiensis* and *Bacillus mycoides*) contain six alkane monooxygenases belonging to three different enzymes. The presence of alkane hydroxylase in multiple copies has been reported in many alkane degraders such as *Alcanivorax borkumensis* SK2 (Sabirova *et al*., 2006), *Pseudomonas aeruginosa* SJTD‐1 (Liu *et al*., 2014) and *Geobacillus thermodenitrificans* NG80‐2 (Liu *et al*., 2009). In addition, *Alcanivorax dieselolei* B‐5 possesses alkane hydroxylase of different systems (Liu *et al*., 2011). However, the *Acinetobacter* species genome is one of the rare cases in terms of alkane degradation repertoire as shown in Fig. 4.

The presence of mobile genetic elements adjacent to alkane hydroxylase in *Rhodococcus* sp. RHA1 and *Azotobacter vinelandii* may suggest that HGT would be one reason for the presence of multiple alkane hydroxylases in a bacterial genome, and this mobile element was also found near the *lad*A1 gene of the DR1 genome (Fig. S6). The existence of *ladA* located in the plasmid of *G. thermodenitrificans* NG80‐2 (pLW1071) also supports the occurrence of horizontal gene transfer. In addition, comparative genomic analysis revealed that DR1 possessed the largest genome among all *Acinetobacter* species probably due to not only gene recombination but also a relatively low rate of gene loss (Jung *et al*., 2011b). Possessing multiple alkane hydroxylase systems in multiple copies may confer ecological advantages for the colonization of niches rich in alkanes.

Increased expression of siderophore biosynthesis‐ and iron transportation‐related genes in DR1 grown on TRI was in concordance with what was observed in *Rhodococcus erythropolis* PR4 (Laczi *et al*., 2015). An increased need for iron might be attributable to high expression of alkane hydroxylase containing di‐iron clusters as reported from *Pseudomonas oleovorans* (Austin *et al*., 2000). In addition, the importance of iron metabolism might be valid especially in marine hydrocarbon‐degrading bacteria. A transposon mutagenesis study of *Alkanivorax borkumenesis* SK2 showed that a kinase sensor *pfeS* regulating iron acquisition by regulating synthesis and secretion of siderophore are essential under UV stress (Sabirova *et al*., 2008).

Several bacterial species, such as the *Rhodococcus* species, secrete various trehalose lipids as a biosurfactant during alkane degradation. A recent report proposed a hypothetical pathway for the synthesis of succinoyl trehalose lipid (STL) based on three essential genes (*fda*,* tlsA* and *alkB*) in *Rhodococcus* sp. SD‐74 during hexadecane degradation (Inaba *et al*., 2013). However, TLC analysis demonstrated similar patterns of glycolipid production in wild type and the *Δots*A mutant strain under TRI, indicating that trehalose synthesis is not necessary for glycolipid production in the DR1 strain (Fig. S7). Another possible hypothesis with respect to trehalose synthesis during TRI metabolism was that of water stress owing to hydrophobicity of the lipophilic substrate (Bhaganna *et al*., 2010). This is based on the highly upregulated *kdp* operon (Table S2), which is also induced under potassium‐limited conditions or osmotic stress (Asha and Gowrishankar, 1993).

The *aceA* gene encoding an isocitrate lyase in glyoxylate bypass was proven to be important for acetate, ethanol, poly‐3‐hydroxybutyrate, alkane metabolisms (Sabirova *et al*., 2006, 2011; Jung *et al*., 2011a; Zhang and Bryant, 2015; Ahn *et al*., 2016; Dunn *et al*., 2009). Interestingly, butanol metabolism appeared to occur through the glyoxylate bypass in *Pseudomonas putida* BIRD‐1 (Cuenca Mdel *et al*., 2016a,b) and loss of ethanol and acetate metabolisms was also observed in *Candida albicans* lacking the isocitrate lyase (Lorenz and Fink, 2001). Alkane degradation generates the TCA cycle intermediates from acetyl‐CoA via the glyoxylate bypass. To confirm the importance of the *aceA* gene during acetate and alkanes metabolisms, growth assays were conducted using wild type and mutant cells. Our data revealed that the *aceA* mutant could not grow on 1% sodium acetate (Fig. S6A); however, the *aceA* mutant could grow on hexadecane and hexadecanoic acid with longer lag phases (Figs S6B and S6C). This unexpected result suggests that DR1 cells might have additional alternative pathways to the glyoxylate shunt during alkane metabolism as seen in *Rhodobacter spaeroedes* and *Methylobacterium extorquens* AM1 (Ensign, 2006). Thus, growth defect of the *Δace*A mutant on TRI might be not due to the limitation in carbon, but due to the sensitivity to the stress generated by TRI. In addition, different pH changes during acetate‐ and hexadecane metabolism suggested that these two substrates are differently metabolized in DR1 cells (Fig. S6D).

**Figure 8:**
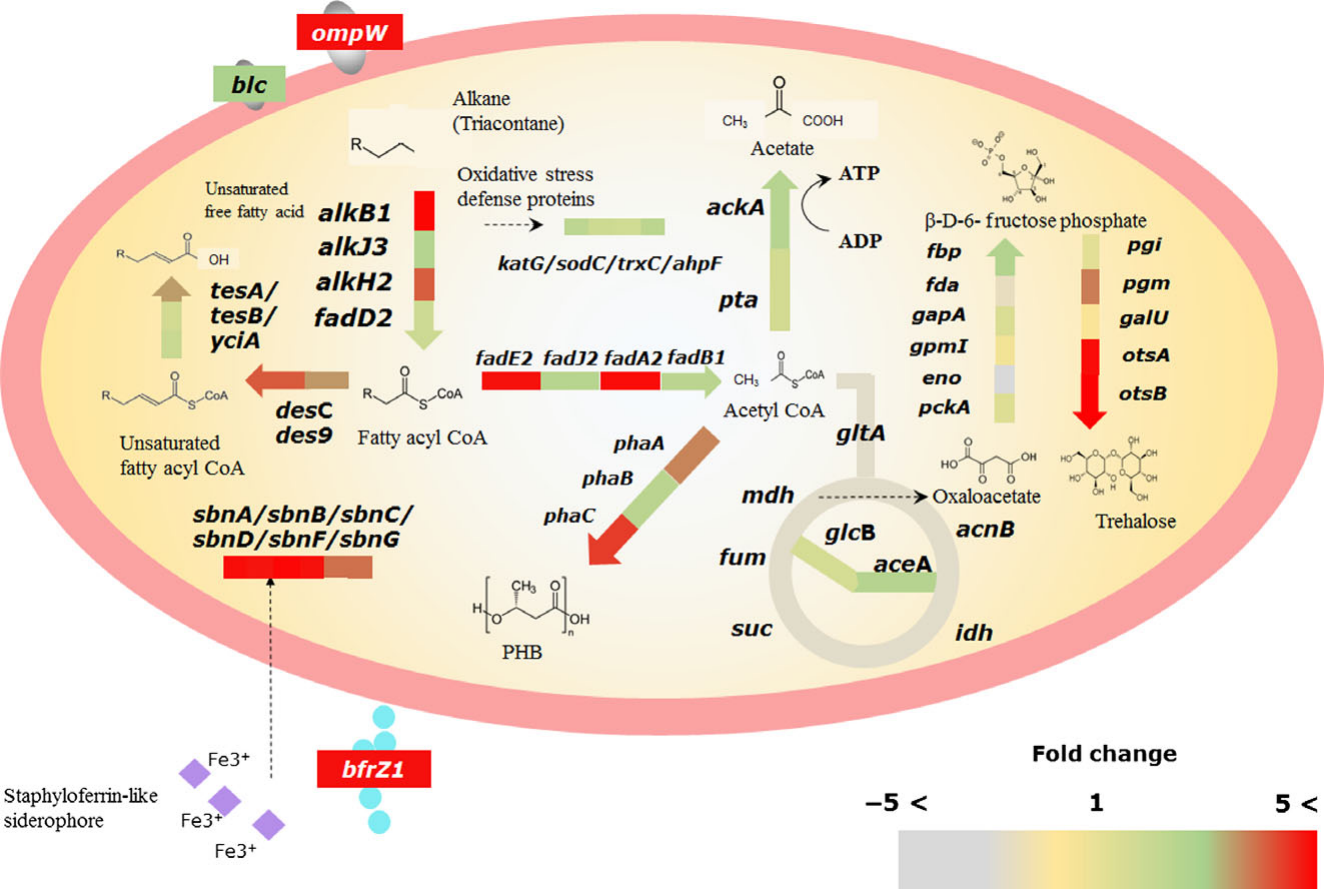
Schematic overview of gene expression within a DR1 cell in the presence of TRI. The colour spectrum indicates a fold change on TRI‐ compared to SUC‐supplemented media. Metabolic pathways were determined using the kegg pathway database and blast comparisons with proteins of experimentally proven function.

It has been reported that outer membrane proteins, such as *ompW*,* ompT*,* oprG* and *blc*, might help to transport alkanes into periplasm of alkane‐degrading bacteria (Sabirova *et al*., 2006, 2011). In total, 42 annotated outer membrane proteins were identified in the DR1 genome (10 receptor proteins, 11 putative outer membrane proteins, 6 lipoproteins and 16 other outer membrane proteins). Both putative alkane transport system protein *ompW* and *blc* were upregulated in the presence of TRI (Table S2, Fig. 8, Fig. S3). Our RNA‐seq data also indicated that many oxidative stress defence genes were highly upregulated in TRI‐amended media (Table S2). All four catalase genes (*kat*Ac, *kat*E, *kat*P, *kat*G) were upregulated, and the *sodC* gene encoding a superoxide dismutase was also induced (Table S2, Fig. 8). Other oxidative stress defence‐related genes (encoding a glutaredoxin, glutaredoxin and alkyl hydroperoxide reductase) were also upregulated by twofold with high RPKM values (> 500) in the presence of TRI (Table S2, Fig. 8). In addition, various general stress response genes (heat‐, cold‐shock protein coding genes, sigma factor, DNA‐repair related genes) were also induced (Table S2). The upregulation of three putative *pha* genes (*pha*A, *pha*B and *pha*C) participating in PHB synthesis led us to confirm the presence of PHB using microscopic analysis with Nile blue A (NBA). More PHB‐accumulated cells were observed with high intensity during TRI utilization (Fig. S8). A recent study revealed that a PHB‐intermediate (methyl esterified 3‐hydroxybutyrate) has hydroxyl radical scavenging activity to protect bacteria from ROS stress (Koskimäki *et al*., 2016). Therefore, upregulation of PHB synthesis genes might be involved in oxidative stress defence.

To our knowledge, this study is the first to evaluate an utilizable alkane range, expression patterns of alkane monooxygenases by chain length of the substrate, and physiological events in *A. oleivorans* DR1 grown on a long‐chain alkane. Notably, expression and mutant studies of alkane monooxygenases provided fundamental information related to the role of the multiple alkane hydroxylation system and regulation of alkane hydroxylases. In addition, RNA‐seq analysis provided valuable data regarding intracellular metabolic and physiological responses to a long‐chain alkane, such as synthesis of PHB, siderophore and trehalose, which were proven experimentally. These data could provide insights into the genotypic and phenotypic appearance of other alkane‐degrading bacteria during the metabolism of hydrophobic substances.

## Experimental procedures

### Bacterial strains, reagents and growth conditions

*Acinetobacter oleivorans* DR1, a diesel degrader, has been characterized in our previous studies (Kang and Park, 2010; Jung *et al*., 2011a; Kim and Park, 2013; Heo *et al*., 2014) and was used in this study. For the growth test of strain DR1, a seed culture was incubated in nutrient broth (Difco, Livonia, MI, USA) at 30°C and 220 r.p.m. overnight. Next, 1 ml of the seed culture was transferred to a 1.7‐ml microtube and washed twice with phosphate‐buffered saline (PBS, pH 7.4). Next, 1 × 10^5^ to 1 × 10^6^ CFU ml^−1^ of strain DR1 was transferred into 20 ml of minimal salt basal (MSB) medium (Hong *et al*., 2014) in a 50‐ml flask to observe the growth on different alkanes [0.1% (v/v for liquid alkanes C_12_–C_22_, w/v for solid alkanes C_24_–C_30_) alkanes]. However, M9 minimal media (Kang *et al*., 2011) was used to conduct only CAS assay for measurement of siderophore production during hexadecane metabolism. Growth curves were generated based on the colony‐forming unit (CFU) owing to water insolubility. All hydrocarbons (*n‐*alkanes) were purchased from Sigma‐Aldrich (St Louis, MO, USA), except for hexane (Junsei, Tokyo, Japan), decane (Wako, Osaka, Japan), decanol (Alfa Aesar, Haverhill, MA, USA), hexadecanol (Daejung, Gyeonggi‐do, Korea) and hexadecanoic acid (Junsei).

### Survival and agar disc diffusion tests

To test the sensitivity of DR1 to decane and decanol, approximately 1 × 10^6^ CFU ml^−1^ of PBS‐washed seed was inoculated into 50 ml fresh MSB media in a 100‐ml flask and the survival test was conducted. After the addition of 0.1% (v/v) decane or decanol, the mixture was shaken gently. Next, a 1 ml sample was transferred to a 1.7‐ml microtube and centrifuged at 1600 × *g* for 1 min. After washing the cell pellet twice, resuspended cells were serially diluted by 10^−1^ to 10^−6^. This procedure was repeated at each time point. Each resuspended sample (100 μl) was then spread onto a nutrient agar plate (NA). Colonies on all plates were counted using a colony doc‐it imaging station (UVP, Upland, CA, USA).

To demonstrate the susceptibility of the wild type and the *otsA* mutant against osmotic stress, a survival test was performed. Exponentially grown wild type and the *otsA* mutant in triacontane supplemented media (~18 h) were harvested and resuspended with 1 ml PBS. Each sample (500 μl) was inoculated into 20 ml PBS containing 5% NaCl. A 1‐ml sample was washed and then diluted with PBS to 10^−4^ at each time point. Diluted samples were spotted onto LB agar plates and the survival rate was calculated:

Survival rate = (CFU ml^−1^ at each time)/(CFU ml^−1^ at 0 h) × 100. For the H_2_O_2_‐sensitivity test of the wild type and *ΔaceA* mutant, overnight‐cultured strains in nutrient broth (NB) were diluted into 50 ml NB by 100‐fold. Exponential‐phase cells (OD_600_~0.6) were harvested and washed twice with PBS. Inoculation of approximately 10^6^ cells per ml was conducted into fresh PBS (10 ml) containing 4 mm H_2_O_2_. At each time point, the cells were harvested and washed in PBS. Viable cells were quantified by counting the CFU. For the agar disc diffusion test, 50 ml MSB agar was autoclaved and cooled for 30 min. Next, 1–1.5 ml of inoculum was transferred into 50 ml MSB agar. The media was shaken gently and poured onto a plate and then dried for another 30 min. An 8‐mm paper disc (Advantec, Dublin, CA, USA) was used to absorb 20 μl of 50 and 100% (v/v) decane or decanol. The decane‐ or decanol‐soaked paper disc was dropped onto a dried MSB agar plate using sterilized forceps.

### RNA isolation and transcriptomic analysis by RNA‐seq

Total RNA of DR1 cells was obtained from mid‐exponentially grown cells using an RNeasy kit (Qiagen, Hilden, Germany) per the manufacturer's instructions. All procedures for RNA sequencing and alignment were conducted by Chunlab (Seoul, South Korea). The Ribo‐Zero rRNA removal kit (Epicentre, Medison, WI, USA) was used for ribosomal RNA depletion according to the manufacturer's instructions. Libraries for Illumina sequencing were constructed with the TruSeq Stranded mRNA sample prep kit (Illumina, San Diego, CA, USA) following the manufacturer's protocol. RNA sequencing was performed on the Illumina HiSeq 2500 platform using single‐end 50‐bp sequencing. Sequence data for the reference genome were retrieved from the NCBI database. Quality‐filtered reads were aligned to the reference‐genome sequence using Bowtie2. The abundance of relative transcript was shown by reads per kilobase of the exon sequence per million mapped sequence reads (RPKM) defined as total exon reads/(mapped reads in millions × exon length in kilobases). Metabolic pathways were analysed based on the kegg pathway analysis and blast alignment with proteins (SRA accession number: SRS1307226).

### Quantitative reverse transcription PCR (qRT‐PCR)

cDNA synthesized from 1 μg of each RNA was used as a template. The PCR mixture contained 1 μl of each primer (0.5 μm) and 2 μl 100‐fold diluted cDNA, 10 μl Power SYBR Green PCR Master Mix (Applied biosystems, Carlsbad, CA, USA) and 6 μl DW comprising a total volume of 20 μl. PCR conditions were set at 48°C for 30 min and 95°C for 10 min for the first holding stage, followed by 42 cycles of 15 s at 95°C for the cycling stage, finally, 15 s at 95°C, 1 min at 60°C and again 15 s at 95°C for the melt curve stage. The expression levels of each gene were normalized by 16S rDNA, as described previously (Watanabe *et al*., 2001). The quantification results were based on triplicate samples. Primer sequences are shown in Table S3.

### Northern blot analysis

Northern blotting was conducted as described previously (Lee *et al*., 2006). Briefly, RNA concentration was measured at OD_260 nm_. Samples of total RNA (1 μl) were loaded onto an ethidium bromide‐stained agarose gel containing 0.25 m formaldehyde and electrophoresed. Separated RNA was transferred to a nylon membrane (Schleicher & Schuell BioScience, Dassel, Germany) using a TurboBlotter. mRNA was quantified by hybridizing the membrane with a P^32^‐labelled probe. Primers used for amplification are represented in Table S3. Autoradiography was conducted using an imaging plate (Fujifilm, Tokyo, Japan) and a multiplex Bio‐imaging system (Fujifilm).

### Phylogenetic analyses of the 16 rRNA gene and alkane monooxygenases

AlkB‐, AlmA‐ and LadA‐type alkane monooxygenase homologs in other bacteria were collected using blastp (http://blast.ncbi.nlm.nih.gov/Blast.cgi). Sequences with over 90% coverage and at least 30% similarity with the query were selected, and bacterial 16S rRNA sequences were searched for in the silva (http://www.arb-silva.de/) web browser. After trimming multiple aligned sequences by clustal w, neighbour‐joining (NJ) phylogenetic trees were drawn using mega 6.0 software. The same criteria were adapted for comparison of alkane homologs between strain DR1 and other bacteria using blastp. The insertion sequence (IS) and mobile genetic element (MGE) were searched using an IS finder (https://www-is.biotoul.fr/) and aclame (http://aclame.ulb.ac.be/).

### Microscopic analysis

Dual staining with NBA and DAPI was performed as previously described (Oshiki *et al*., 2013). Briefly, heat‐fixed cells were treated with 1% ethanolic Nile blue A (NBA) for 30 min to visualize the intracellular granules accumulated by PHA. After washing with 100% ethanol, DR1 cells were stained with 2 μg ml^−1^ DAPI for 5 min. NBA‐stained granules and DAPI‐stained cells were detected as bright red and blue at 550 nm‐, 365 nm wavelength excitation filters respectively.

### Chrome azurol S (CAS) assay

To quantify the production of siderophore, the CAS assay was conducted as previously described (Dale *et al*., 2004). Briefly, all 1/10 diluted supernatants and a blank were mixed with equal volumes of CAS shuttle solution and then allowed to stand for 30 min at room temperature. The absorbance at 630 nm was determined with MSB as the blank and distilled water (DW) as the reference standard. Siderophore units were measured as follows: (A_630_ of MSB−A_630_ of sample)/A_630_ of MSB × 100% and were standardized by cell density (OD_600_).

### Trehalose assay

Supernatant and crude cells grown on 50 ml MSB media were separated by ultracentrifugation (3000 r.p.m., 20 min). Then, intracellular trehalose was extracted by boiling the cell pellet at 90°C for 20 min. Trehalose levels of supernatant and crude cells were measured using a Trehalose assay kit K‐TREH (Megazyme International Ireland, Bray, Ireland) according to the manufacturer's instructions. All samples were standardized by the Bradford assay, and triplicates were used.

### ATP assay

Measurement of intracellular ATP concentration was conducted using the ENLITEN ATP Assay System Bioluminescence Detection Kit (Promega, Medison, WI, USA) in accordance with the manufacturer's instructions. Briefly, exponentially cultured DR1 and *ΔackA* KO mutant were harvested and resuspended in 1% trichloroacetic acid (TCA) buffer. Prior to the measurement, the samples were neutralized by sixfold dilution with 250 mm Tris–acetate buffer (pH 7.75). The luminescence was measured using a microplate reader (Hidex, Turku, Finland).

### Construction of the single crossover knockout (KO) mutants

The *alkR1* (AOLE_RS10595), *alkR2* (AOLE_RS13405), *alkB1* (AOLE_RS10590), *alkB2* (AOLE_RS13400), *otsA* (AOLE_RS15640), *ackA* (AOLE_RS17025) and *aceA* (AOLE_RS14285) genes were amplified by PCR from genomic DNA and cloned into the pVIK 112 plasmid. The amplified fragments were digested with KpnI/XbaI for *alkB1*,* alkB2* and *aceA*, and EcoRI/KpnI for *otsA* and *ackA*. Ligation into each of the restriction enzyme‐treated sites of pVIK112 was performed; the plasmids were subsequently transformed into the *E. coli* S17‐1λ pir strain. Recombined plasmids were extracted and then transformed to strain DR1. KO mutants were screened on NA containing 50 μg ml^−1^ kanamycin.

To produce an *alkB* double KO mutant, *alkB2* amplicon and pEX18Gm vector were treated with the same restriction enzymes as above. After ligation, the recombined vector was transformed into the *E. coli* S17‐1λ pir strain. The vector obtained by extraction from *E. coli* was again transformed into strain *alkB1* single KO strain, and then, the double KO mutant was selected on NA containing 50 μg ml^−1^ kanamycin and 15 μg ml^−1^ gentamicin.

To construct an *aceA* complemented strain, the amplified fragment of *aceA* and pRK415 vector was digested with BamHI/EcoRI. The constructed plasmid was transformed into the *E. coli* TOP10. Complementation was performed by transforming this constructed vector into *aceA* KO strain, which was screened on LB containing 50 μg ml^−1^ kanamycin and 20 μg ml^−1^ tetracycline. Gel electrophoresis and sequencing were conducted with KO_F, KO_R, and KO_C primers for verification (Table S3).

### Thin‐layer chromatography (TLC) analysis

Glycolipid of the wild type and mutant strain was analysed by thin‐layer chromatography as previously described (Espuny *et al*., 1995). Briefly, mid‐exponentially grown cells in 50 ml MSB medium were harvested by ultracentrifugation. Cells collected were resuspended in 5 ml PBS and brought to pH 2 using H_2_SO_4_. Then, 5 ml of the mixture (chloroform: methanol = 2:1) was added and vortexed for 5 min. The organic phase was dried in a rotary evaporator and resuspended in 40 μl fresh chloroform/methanol mixture. Samples were plated on silica gel sheets G 60 (Merck, Kenilworth, NJ, USA) and developed with chloroform/methanol/water = 65:25:4). Visualization of developed samples was performed by treatment with TLC reagents: iodine vapour for lipid staining and 1‐naphthol reagent for hydrocarbon detection (Wang and Benning, 2011).

## Conflict of interest

None declared.

## Supporting information


**Table S1.** Information about the raw RNA‐seq used in this study.
**Table S2.** RPKM values of transcriptome in DR1 strain under SUC and TRI. Fold changes were determined by RPKM values of each gene under TRI compared to SUC.
**Table S3.** Information about primer sequences used in qRT‐PCR, Northern blotting, and construction of knock out mutants.
**Fig. S1.** Growth assays and survival tests on (A) hexane, (B) decane, and their derivatives. (C) Measurement of colony‐forming units (CFUs) for 24 h to verify the inability of cells to grow on 0.1% hexane and decane. All experiments were performed in triplicate and their means are represented. (D) Survival tests on 0.1% decanol within 15 min was performed for *A. oleivorans* DR1. (E) Verification of decanol‐high toxicity towards DR1 in a paper disk assay. The results of the paper disk assay on decane (top) and decanol (bottom) are shown as 50% (left) and 100% (right).
**Fig. S2.** The expression analysis of *alkB* in wild type strain and *alkB* single mutants using Northern blot hybridization. (A) *alkB1* expression in wild type‐, and *ΔalkB2 strain*. (B) *alkB2* expression in wild type‐, and *ΔalkB1 strain*.
**Fig. S3.** Validation of six‐upregulated genes in RNA‐seq profile using qRT‐PCR.
**Fig. S4.** (A) CAS activity and (B) relative expression level of *sbnA* in DR1 cells grown on 10 mm succinate (SUC, cyan), and 0.1% hexadecane (HEX, red) supplemented M9 medium.
**Fig. S5.** The scheme of MGE site in the upstream of *ladA1* in DR1 strain and *tnpA*‐encoding genes in *Azotobacter vinelandii*.
**Fig. S6.** Growth assay of wild type‐, *ΔaceA‐, ΔaceA*(pRK415::*aceA*) strain on (A) 1% Sodium acetic acid, (B) 1% hexadecane, (C) 1% hexadecanoic acid. (D) pH measurement of wild type strain during sodium acetic acid (NaAc, red), and hexadecane (HEX, green) assimilation. Circle and square indicates OD_600_ and pH, respectively.
**Fig. S7.** Comparison of intracellular glycolipid between wild type and *ΔotsA* KO strain using thin‐layer chromatography (TLC). Numbers beside the column indicate the *R*_f_ value of each band. Left side indicates detection of hydrocarbon in glycolipid using 1‐naphthol reagent. Right side indicates detection of lipid in glycolipid using iodine vapor.
**Fig. S8.** Microscopic analysis of NBA‐, DAPI‐ dual stained DR1 for PHB detection. Mid‐exponential cells were stained and observed by no filter (control), 365 nm‐ (DAPI), and 550 nm‐wavelength filters (NBA). Merged images of DAPI and NBA are shown on the right side (DAPI + NBA).
