# Supplementary material for: Metabolic and stress responses of Acinetobacter oleivorans DR1 during long‐chain alkane degradation
Source: Microb Biotechnol. 2017 Aug 31;10(6):1809–23. doi: 10.1111/1751-7915.12852 (PMC5658608; doi:10.1111/1751-7915.12852)
Supplement: Supplementary file 1 — Table S1. Information about the raw RNA‐seq used in this study. Table S2. RPKM values of transcriptome in DR1 strain under SUC and TRI. Fold changes were determined by RPKM values of each gene under TRI compared to SUC. Table S3. Information about primer sequences used in qRT‐PCR, Northern blotting, and construction of knock out mutants. Fig. S1. Growth assays and survival tests on (A) hexane, (B) decane, and their derivatives. (C) Measurement of colony‐forming units (CFUs) for 24 h to verify the inability of cells to grow on 0.1% hexane and decane. All experiments were performed in triplicate and their means are represented. (D) Survival tests on 0.1% decanol within 15 min was performed for A. oleivorans DR1. (E) Verification of decanol‐high toxicity towards DR1 in a paper disk assay. The results of the paper disk assay on decane (top) and decanol (bottom) are shown as 50% (left) and 100% (right). Fig. S2. The expression analysis of alkB in wild type strain and alkB single mutants using Northern blot hybridization. (A) alkB1 expression in wild type‐, and ΔalkB2 strain. (B) alkB2 expression in wild type‐, and ΔalkB1 strain. Fig. S3. Validation of six‐upregulated genes in RNA‐seq profile using qRT‐PCR. Fig. S4. (A) CAS activity and (B) relative expression level of sbnA in DR1 cells grown on 10 mm succinate (SUC, cyan), and 0.1% hexadecane (HEX, red) supplemented M9 medium. Fig. S5. The scheme of MGE site in the upstream of ladA1 in DR1 strain and tnpA‐encoding genes in Azotobacter vinelandii. Fig. S6. Growth assay of wild type‐, ΔaceA‐, ΔaceA(pRK415::aceA) strain on (A) 1% Sodium acetic acid, (B) 1% hexadecane, (C) 1% hexadecanoic acid. (D) pH measurement of wild type strain during sodium acetic acid (NaAc, red), and hexadecane (HEX, green) assimilation. Circle and square indicates OD600 and pH, respectively. Fig. S7. Comparison of intracellular glycolipid between wild type and ΔotsA KO strain using thin‐layer chromatography (TLC). Numbers beside the column indicate [file MBT2-10-1809-s001.docx]

**Table S1** Information about the raw RNA-seq used in this study.

|  | **SUC** | **TRI** |
| --- | --- | --- |
| Total reads | 29,815,913 | 31,232,629 |
| Total mapped reads (%) | 95.9 | 95.5 |
| Average RPKM | 314.6 | 372.4 |
| Median RPKM | 58.7 | 77.1 |

**Table S2** RPKM values of transcriptome in DR1 strain under SUC and TRI. Fold changes were determined by RPKM values of each gene under TRI compared to SUC.

| Gene product | Symbol | | Locus tag | | SUC | | TRI | | Fold change | | |
| --- | --- | --- | --- | --- | --- | --- | --- | --- | --- | --- | --- |
| Alkane metabolism | | | | | | | | | | | |
| Alkane hydroxylase | *alkB1* | | AOLE_RS10590 | | 291.3 | | 3387.4 | | 11.6 | | |
|  | *alkB2* | | AOLE_RS13400 | | 512.2 | | 3694.0 | | 7.2 | | |
|  | *almA1* | | AOLE_RS02255 | | 145.4 | | 223.4 | | 1.5 | | |
|  | *almA2* | | AOLE_RS09555 | | 10.0 | | 56.7 | | 5.7 | | |
|  | *ladA1* | | AOLE_RS11290 | | 64.8 | | 27.2 | | 0.4 | | |
|  | *ladA2* | | AOLE_RS11525 | | 8.0 | | 22.3 | | 2.8 | | |
|  | *alkG* | | AOLE_RS14345 | | 1337.8 | | 1271.8 | | 1.0 | | |
|  | *alkT* | | AOLE_RS14350 | | 394.5 | | 294.1 | | 0.7 | | |
| Alkane hydroxylase regulator | *alkR1* | | AOLE_RS10595 | | 47.6 | | 121.6 | | 2.6 | | |
|  | *alkR2* | | AOLE_RS13405 | | 34.9 | | 96.2 | | 2.8 | | |
| Alcohol dehydrogenase | *alkJ3* | | AOLE_RS08985 | | 58.4 | | 126.7 | | 2.2 | | |
|  | *alkJ4* | | AOLE_RS09270 | | 24.8 | | 40.7 | | 1.6 | | |
| Aldehyde dehydrogenase | *alkH2* | | AOLE_RS17195 | | 135.3 | | 600.9 | | 4.4 | | |
|  | *alkH3* | | AOLE_RS06800 | | 3445.5 | | 4393.4 | | 1.3 | | |
| Fatty acid β-oxidation | | | | | | | | | | | |
| Acyl-CoA synthetase | *fadD1* | | AOLE_RS11865 | | 24.8 | | 23.6 | | 1.0 | | |
|  | *fadD2* | | AOLE_RS16535 | | 111.4 | | 143.4 | | 1.3 | | |
| Acyl-CoA dehydrogenase | *fadE1* | | AOLE_RS11950 | | 19.4 | | 20.4 | | 1.1 | | |
|  | *fadE2* | | AOLE_RS13150 | | 54.2 | | 488.0 | | 9.0 | | |
|  | *fadE3* | | AOLE_RS10580 | | 58.2 | | 327.5 | | 5.6 | | |
|  | *fadE4* | | AOLE_RS10585 | | 47.1 | | 214.3 | | 4.6 | | |
| Enoyl-CoA hydratase | *fadJ1* | | AOLE_RS18860 | | 45.8 | | 18.6 | | 0.4 | | |
|  | *fadJ2* | | AOLE_RS14620 | | 221.8 | | 391.2 | | 1.8 | | |
| 3-hydroxy-  acyl-CoA dehydrogenase | *fadB1* | | AOLE_RS11965 | | 9.3 | | 17.6 | | 1.9 | | |
|  | *fadB2* | | AOLE_RS17935 | | 1015.4 | | 540.5 | | 0.5 | | |
| 3-ketoacyl-CoA thiolase | *fadA1* | | AOLE_RS17940 | | 913.4 | | 327.0 | | 0.4 | | |
|  | *fadA2* | | AOLE_RS08510 | | 17.6 | | 116.7 | | 6.6 | | |
|  | *fadA3* | | AOLE_RS07055 | | 820.0 | | 2122.6 | | 2.6 | | |
| Synthesis of free unsaturated fatty acid | | | | | | | | | | | |
| Stearoyl-CoA desaturase | *desC* | | AOLE_RS02930 | | 1546.9 | | 4908.0 | | 3.2 | | |
| Delta-9 desaturase | *des9* | | AOLE_RS12765 | | 12.4 | | 34.2 | | 2.8 | | |
| Acyl-CoA thioesterase | *tesA* | | AOLE_RS14395 | | 49.1 | | 126.9 | | 2.6 | | |
|  | *tesB* | | AOLE_RS02070 | | 228.5 | | 286.6 | | 1.3 | | |
|  | *yciA* | | AOLE_RS02685 | | 250.4 | | 388.3 | | 1.6 | | |
| Synthesis of Poly(3-hydroxybutyrate) (PHB) | | | | | | | | | | | |
| Acetyl-CoA acetyltransferase | *phaA* | | AOLE_RS07055 | | 820.0 | | 2122.6 | | 2.6 | | |
| 3-ketoacyl-acyl carrier protein (ACP) reductase | *phaB* | | AOLE_RS07060 | | 616.2 | | 895.4 | | 1.5 | | |
| Poly(3-hydroxyalkanoate) synthetase | *phaC* | | AOLE_RS06095 | | 49.3 | | 221.7 | | 4.5 | | |
| Oxidative stress defense | | | | | | | | | | | |
| Regulator | *oxyR* | | AOLE_RS14360 | | 284.7 | | 525.5 | | 1.8 | | |
|  | *soxR* | | AOLE_RS12160 | | 8.3 | | 35.9 | | 4.3 | | |
| Catalase | *katAc* | | AOLE_RS09860 | | 45.1 | | 362.6 | | 8.0 | | |
|  | *katE* | | AOLE_RS11805 | | 24.1 | | 815.4 | | 33.8 | | |
|  | *katP* | | AOLE_RS12765 | | 50.2 | | 67.0 | | 1.3 | | |
|  | *katG* | | AOLE_RS17405 | | 5482.2 | | 12274.2 | | 2.2 | | |
| Superoxide dismutase | *sodC* | | AOLE_RS01790 | | 406.3 | | 751.6 | | 1.8 | | |
|  | *sodB* | | AOLE_RS05430 | | 6311.4 | | 3753.9 | | 0.6 | | |
| Glutaredoxin | *grxD* | | AOLE_RS08240 | | 1011.5 | | 1656.4 | | 1.6 | | |
|  | *grxC* | | AOLE_RS16810 | | 1217.0 | | 1589.2 | | 1.3 | | |
| Thioredoxin | *trxC* | | AOLE_RS02680 | | 326.8 | | 554.4 | | 1.7 | | |
|  | *trxB* | | AOLE_RS15310 | | 1575.4 | | 1996.0 | | 1.3 | | |
|  | *trxA* | | AOLE_RS16455 | | 5856.8 | | 7822.2 | | 1.3 | | |
| Alkyl hydroperoxide reductase | *ahpF1* | | AOLE_RS11430 | | 9150.4 | | 18062.0 | | | | 2.0 |
|  | *ahpF2* | | AOLE_RS13420 | | 8438.9 | | 15310.7 | | | | 1.8 |
|  | *ahpC* | | AOLE_RS13390 | | 22479.9 | | 47977.9 | | | | 2.1 |
| Iron uptake | | | | | | | | | | | |
| Siderophore biosynthesis | *sbnG* | | AOLE_RS07230 | | 8.2 | | 24.2 | | 3.0 | | |
|  | *sbnF* | | AOLE_RS07235 | | 7.5 | | 24.6 | | 3.3 | | |
|  | *sbnD* | | AOLE_RS07240 | | 5.3 | | 28.6 | | 5.4 | | |
|  | *sbnC* | | AOLE_RS07245 | | 15.7 | | 108.2 | | 6.9 | | |
|  | *sbnB* | | AOLE_RS07250 | | 36.7 | | 226.9 | | 6.2 | | |
|  | *sbnA* | | AOLE_RS07255 | | 57.0 | | 595.5 | | 10.4 | | |
| Siderophore receptor | *bfrZ1* | | AOLE_RS10495 | | 13.3 | | 228.3 | | 17.1 | | |
|  | *bfrZ2* | | AOLE_RS12695 | | 37.0 | | 78.8 | | 2.1 | | |
|  | *bfrZ3* | | AOLE_RS17065 | | 31.8 | | 102.9 | | 3.2 | | |
| Gluconeogenesis | | | | | | | | | | | |
| Phosphoenolpyruvate  carboxykinase | *pckA* | | AOLE_RS04025 | | 870.5 | | 876.1 | | 1.0 | | |
| Enolase | *eno* | | AOLE_RS08460. | | 1003.4 | | 307.3 | | 0.3 | | |
| Phosphoglyceromutase | *gpmI* | | AOLE_RS18295 | | 402.5 | | 316.3 | | 0.8 | | |
| Glyceraldehyde  3-phosphate  dehydrogenase | *gapA* | | AOLE_RS13565 | | 57.3 | | 62.8 | | 1.1 | | |
| Fructose-1,6-bisphosphate aldolase | *fda* | | AOLE_RS10955 | | 1401.8 | | 643.4 | | 0.5 | | |
| Fructose-1,6-bisphosphatase | *fbp* | | AOLE_RS04465 | | 142.9 | | 270.0 | | 1.9 | | |
| Trehalose synthesis | | | | | | | | | | | |
| Glucose-6-phosphate isomerase | *pgi* | | AOLE_RS19115 | | 215.3 | | 198.3 | | 0.9 | | |
| Phosphomannomutase  /phosphoglucomutase | *pgm* | | AOLE_RS15250 | | 211.7 | | 640.0 | | 3.0 | | |
| UTP-glucose-1-phosphate uridylyltransferase | *galU* | | AOLE_RS19125 | | 381.5 | | 270.5 | | 0.7 | | |
| Alpha,alpha-trehalose-phosphate synthase | *otsA* | | AOLE_RS15640 | | 25.8 | | 686.5 | | 26.6 | | |
| Trehalose-hosphatase | *otsB* | | AOLE_RS15635 | | 4.2 | | 487.6 | | 116.4 | | |
| TCA cycle and glyoxylate shunt | | | | | | | | | | | |
| Citrate synthase | *gltA* | | AOLE_RS03800 | | 1723.8 | | 831.0 | | 0.5 | | |
| Aconitate hydratase | *acnB1* | | AOLE_RS06655 | | 599.5 | | 538.7 | | 0.9 | | |
|  | *acnB2* | | AOLE_RS16695 | | 529.8 | | 707.9 | | 1.3 | | |
|  | *acnB3* | | AOLE_RS19050 | | 299.8 | | 67.9 | | 0.2 | | |
| Isocitrate dehydrogenase | *idh1* | | AOLE_RS04880 | | 1335.0 | | 457.3 | | 0.3 | | |
|  | *idh2* | | AOLE_RS04890 | | 146.6 | | 83.3 | | 0.6 | | |
| 2-Oxoglutarate dehydrogenase | *sucA* | | AOLE_RS03830 | | 531.3 | | 569.4 | | 1.1 | | |
| Dihydrolipoyllysine-residue succinyltransferase | *sucB* | | AOLE_RS03835 | | 936.5 | | 278.1 | | 0.3 | | |
| Succinyl-CoA synthetase | *sucCb* | | AOLE_RS03845 | | 751.2 | | 68.1 | | 0.1 | | |
|  | *sucCa* | | AOLE_RS03850 | | 1478.9 | | 152.6 | | 0.1 | | |
| Succinate dehydrogenase | *sdhC* | | AOLE_RS03805 | | 1187.1 | | 950.8 | | 0.8 | | |
|  | *sdhD* | | AOLE_RS03810 | | 1556.0 | | 995.0 | | 0.6 | | |
|  | *sdhA* | | AOLE_RS03815 | | 1703.7 | | 638.2 | | 0.4 | | |
|  | *sdhB* | | AOLE_RS03820 | | 2158.2 | | 555.8 | | 0.3 | | |
| Fumarate hydratase | *fumC* | | AOLE_RS07365 | | 867.4 | | 835.2 | | 1.0 | | |
| Fumarase | *fumB* | | AOLE_RS17035 | | 458.7 | | 371.0 | | 0.8 | | |
| Malate dehydrogenase | *mdh* | | AOLE_RS02410 | | 2467.1 | | 754.4 | | 0.3 | | |
| Isocitrate lyase | *aceA* | | AOLE_RS14285 | | 1193.7 | | 3138.3 | | 2.6 | | |
| Malate synthase G | *glcB* | | AOLE_RS10780 | | 874.8 | | 968.0 | | 1.1 | | |
| Energy metabolism | | | | | | | | | | | |
| F0F1 ATP synthase | *atpC* | | AOLE_RS18620 | | 6023.2 | | 818.5 | | 0.1 | | |
|  | *atpD* | | AOLE_RS18625 | | 6608.7 | | 693.3 | | 0.1 | | |
|  | *atpG* | | AOLE_RS18630 | | 5581.1 | | 640.2 | | 0.1 | | |
|  | *atpA* | | AOLE_RS18635 | | 5799.3 | | 637.8 | | 0.1 | | |
|  | *atpH* | | AOLE_RS18640 | | 4125.1 | | 608.2 | | 0.1 | | |
|  | *atpF* | | AOLE_RS18645 | | 2630.4 | | 329.9 | | 0.1 | | |
|  | *atpE* | | AOLE_RS18650 | | 8507.4 | | 1084.6 | | 0.1 | | |
|  | *atpB* | | AOLE_RS18655 | | 2005.2 | | 579.7 | | 0.3 | | |
| Acetate kinase | *ackA* | | AOLE_RS17025 | | 256.8 | | 716.5 | | 2.8 | | |
| Phosphate acetyl transferase | *pta* | | AOLE_RS17025 | | 147.5 | | 196.0 | | 1.3 | | |
| Adaptation of cell surface | | | | | | | | | | | |
| Alkane transporter | *ompW* | | AOLE_RS05520 | | 12.0 | | 181.3 | | 15.2 | | |
|  | *blc1* | | AOLE_RS04765 | | 247.2 | | 455.0 | | 1.8 | | |
|  | *blc2* | | AOLE_RS06180 | | 75.9 | | 169.7 | | 2.2 | | |
| PNAG Cluster I | *pgaD1* | | AOLE_RS14635 | | 64.9 | | 90.4 | | 1.4 | | |
|  | *pgaC1* | | AOLE_RS14640 | | 71.4 | | 89.3 | | 1.3 | | |
|  | *pgaB1* | | AOLE_RS14645 | | 54.2 | | 78.1 | | 1.4 | | |
|  | *pgaA1* | | AOLE_RS14650 | | 116.0 | | 191.4 | | 1.6 | | |
| PNAG cluster II | *pgaA2* | | AOLE_RS06490 | | 7.7 | | 17.0 | | 2.2 | | |
|  | *pgaB2* | | AOLE_RS06495 | | 13.8 | | 15.8 | | 1.2 | | |
|  | *pgaC2* | | AOLE_RS06500 | | 17.3 | | 34.4 | | 2.0 | | |
|  | *pgaD2* | | AOLE_RS06505 | | 47.1 | | 91.62 | | 1.9 | | |
| Potassium limitation (osmotic stress) response | | | | | | | | | | | |
| Potassium transporting ATPase | *kdpA* | | AOLE_RS06590 | | 3.8 | | 341.1 | | 91.0 | | |
|  | *kdpB* | | AOLE_RS06595 | | 3.9 | | 150.6 | | 38.3 | | |
|  | *kdpC* | | AOLE_RS06600 | | 6.2 | | 140.3 | | 22.7 | | |
| Histine kinase | *kdpD* | | AOLE_RS06605 | | 25.4 | | 82.6 | | 3.3 | | |
| Response regulator | *kdpE* | | AOLE_RS06610 | | 32.6 | | 59.5 | | 1.8 | | |
| General stress response genes | | | | | | | | | | | |
| DNA polymerase | | *umuD* | | AOLE_RS11780 | | 80.4 | | 134.1 | | 1.7 | |
| RNA polymerase  sigma factor | | *rpoS1* | | AOLE_RS03770 | | 1118.9 | | 1969.7 | | 1.8 | |
|  |  | *rpoS2* | | AOLE_RS05865 | | 753.8 | | 2126.9 | | 2.8 | |
| Cold shock protein | | *cspG1* | | AOLE_RS05230 | | 38.0 | | 130.9 | | 3.4 | |
|  |  | *cspG2* | | AOLE_RS05875 | | 4383.6 | | 4615.6 | | 1.1 | |
|  |  | *cspG3* | | AOLE_RS06005 | | 3306.4 | | 13623.2 | | 4.1 | |
| Heat shock protein | | *hsp1* | | AOLE_RS17980 | | 323.7 | | 442.8 | | 1.4 | |
|  |  | *hsp2* | | AOLE_RS07505 | | 120.5 | | 845.8 | | 7.0 | |
|  |  | *htp* | | AOLE_RS17980 | | 1106.1 | | 1886.4 | | 1.7 | |
| Recombinase | | *recA* | | AOLE_RS07500 | | 1086.0 | | 1092.4 | | 1.0 | |
| Excinuclease | | *uvrA* | | AOLE_RS00930 | | 251.65 | | 273.72 | | 1.1 | |
|  |  | *uvrB* | | AOLE_RS04760 | | 183.95 | | 392.58 | | 2.1 | |
|  |  | *uvrC* | | AOLE_RS17910 | | 55.98 | | 118.7 | | 2.1 | |
| Endopeptidase | | *nlpD* | | AOLE_RS13515 | | 95.9 | | 108.3 | | 1.1 | |
| Endonuclease | | *nth* | | AOLE_RS14205 | | 39.3 | | 112.1 | | 2.9 | |
| DNA-glycosylase | | *ung* | | AOLE_RS10845 | | 131.0 | | 203.3 | | 1.6 | |
|  |  | *mutM* | | AOLE_RS03160 | | 92.1 | | 191.4 | | 2.1 | |

**Table S3** Information about primer sequences used in qRT-PCR, Northern blotting, and construction of knock out mutants.

| **Primer** | **Sequences (5' - 3')** | |
| --- | --- | --- |
| **qRT-PCR** | | |
| *ackA*_F | TTCCATCAGACCATGCCACC | |
| *ackA*_R | CCTAGATGCGCTGTTAGCCA | |
| *otsA*_F | GGCGACCTCTTCCGTGTTTA | |
| *otsA*_R | CGGGGCTGGTTTCCAAAAAG | |
| *ompW*_F | CGCGGTCTAAGTCAATGGGA | |
| *ompW*_R | TTGCTGTGGCTGAAAAAGGC | |
| *sbnA*_F | AACAACTGCCAAATGCCGTC | |
| *sbnA*_R | CGAGACACGCCCATAATCGT | |
| *phaA*_F | CGCCGATTGCTGTAGGTGAT | |
| *phaA*_R | CGTGGTTCACCATTGCTTGG | |
| *bfrZ*_F | CCCAATGCAAACAACTGGGG | |
| *bfrZ*_R | CAGCTTAGCGGTCCACTGAT | |
| *alkB1*_F | GCCTGCGTCTATGCGTGAAAT | |
| *alkB1*_R | CAAAGGACGTGGACCAAACT | |
| *alkB2*_F | GTCGAGCAAGCGACAACAAC | |
| *alkB2*_R | TGCTAACCCACCCATTGCA | |
| *almA1*_F | TGCGTGAAGGTAAAGCGAGT | |
| *almA1*_R | TAGCCTGAACCCCACCCATA | |
| *almA2*_F | CGACCCTATTGCACTGGCTT | |
| *almA2*_R | AGTCGCCGTTTTCCAGATGT | |
| **Knock out** | | |
| *alkB1*_F | CGCGGTACCTGGCCACGTACTGTTGTTGG | |
| *alkB1*_R | CGCTCTAGATAGCTCAGGTGCCTCGTCAA | |
| *alkB2*_F | CGCGGTACCTTGCAATGGGTGGGTTAGCA | |
| *alkB2*_R | CGCTCTAGACGGTGGTGTCCATAAGGGTG | |
| *otsA*_F | CGCGAATTCGGCGACCTCTTCCGTGTTTA | |
| *otsA*_R | CGCGGTACCCCATCGCGTAGTGAGCTGAT | |
| *ackA*_F | CGCGAATTCCTCCGCTTCATAACCCAGCA | |
| *ackA*_R | CGCGGTACCACGCCTTCAAGTGGAGTGAG | |
| *aceA*_F | CGCCCCGGGCGAAACTGCGACTCCAAACG | |
| *aceA*_R | CGCGGTACCGTACGCCAGCAACATAAGCC | |
| **Northern blot** | | |
| *alkB1*_F | | TCTGGCCACGTACTGTTGTT |
| *alkB1*_R | | GCTTGGAATGGACGAGTCGG- |
| *alkB2*_F | | TACGGCACATGAACTCAGCC |
| *alkB2*_R | | CGCCGATTTAAAGCTGCCAA |
| *almA1*_F | | TAAGCCGTGGTGTAAAGCGA |
| *almA1*_R | | TCGCCATTGCCGGTACTAAA |
| *almA2*_F | | TTCCTGCTTATGGGCACAGG |
| *almA2*_R | | ACATCTGGAAAACGGCGACT |
| **Complementation** | | |
| *aceA_com*_F | | CGCGGATCCTGAAGGCTCTTTAGCTGAGC |
| *aceA_com*_R | | CGCGAATTCGAGTTACCCCAAGCTGTTCG |

**Fig. S1**

**
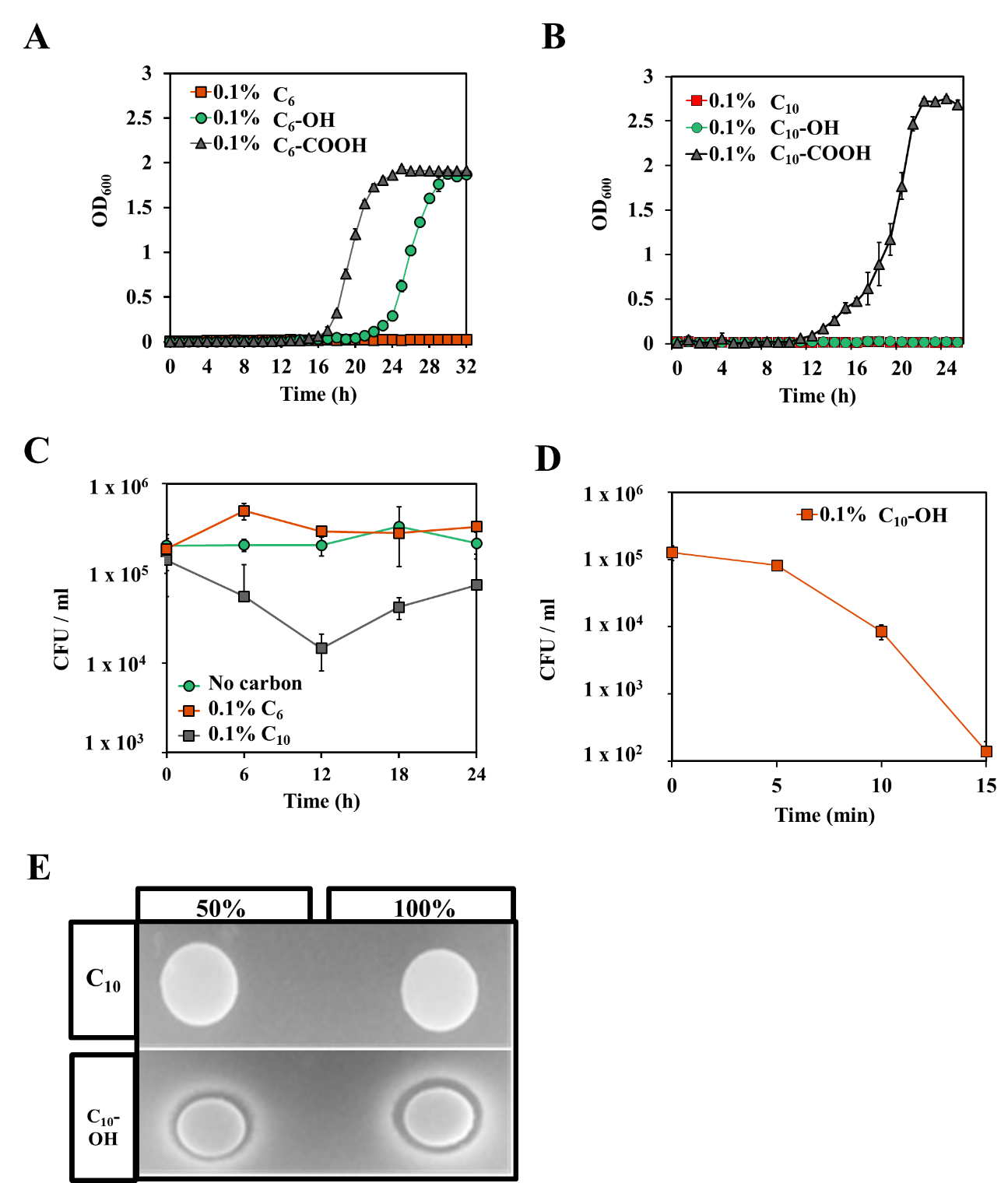
**

**Fig. S2**

**
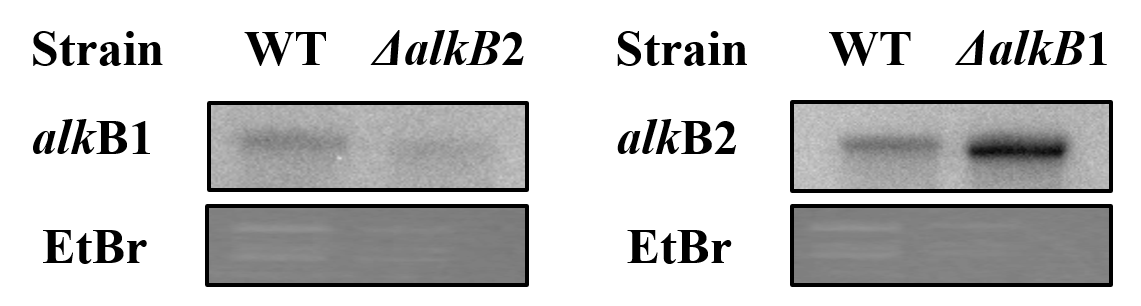
**

**Fig. S3**

**
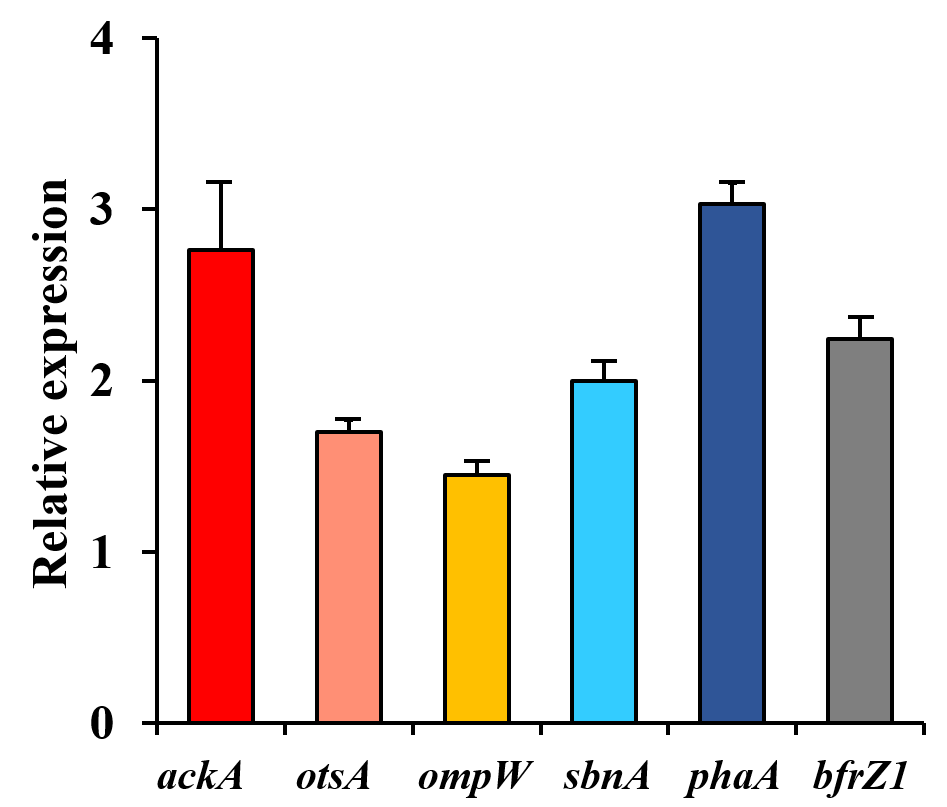
**

**Fig. S4**

**
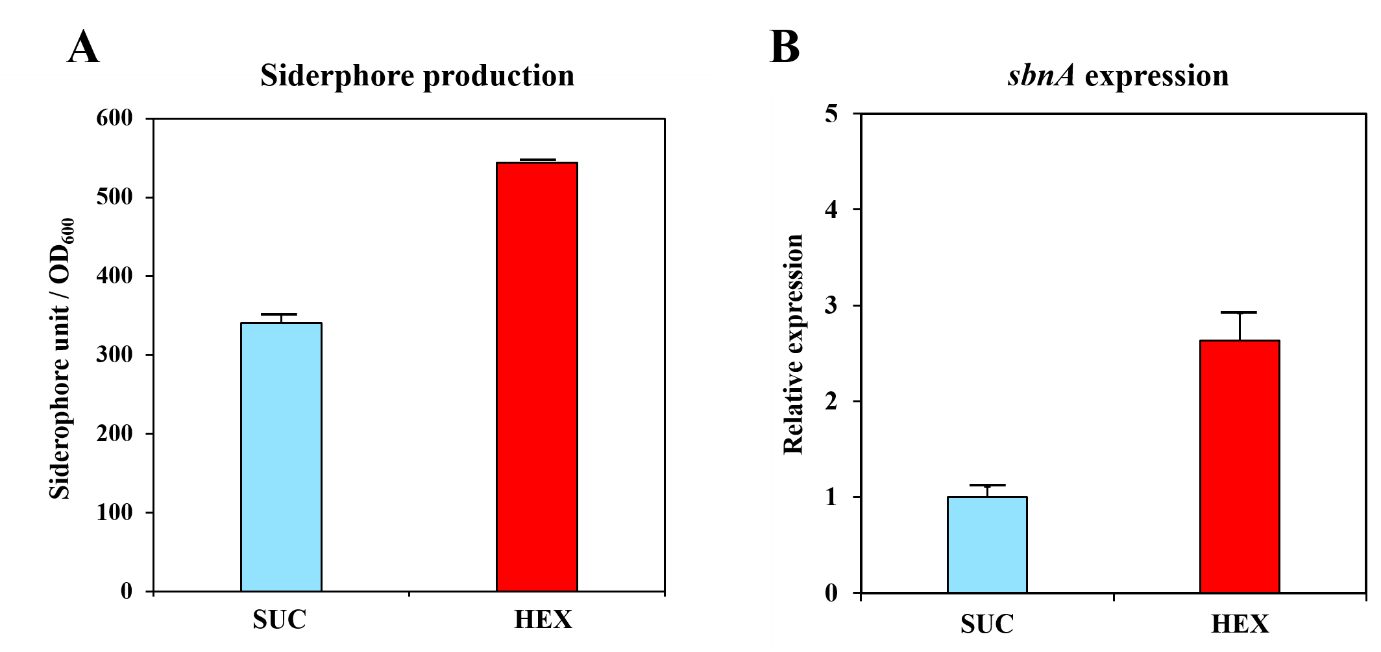
**

**Fig. S5**


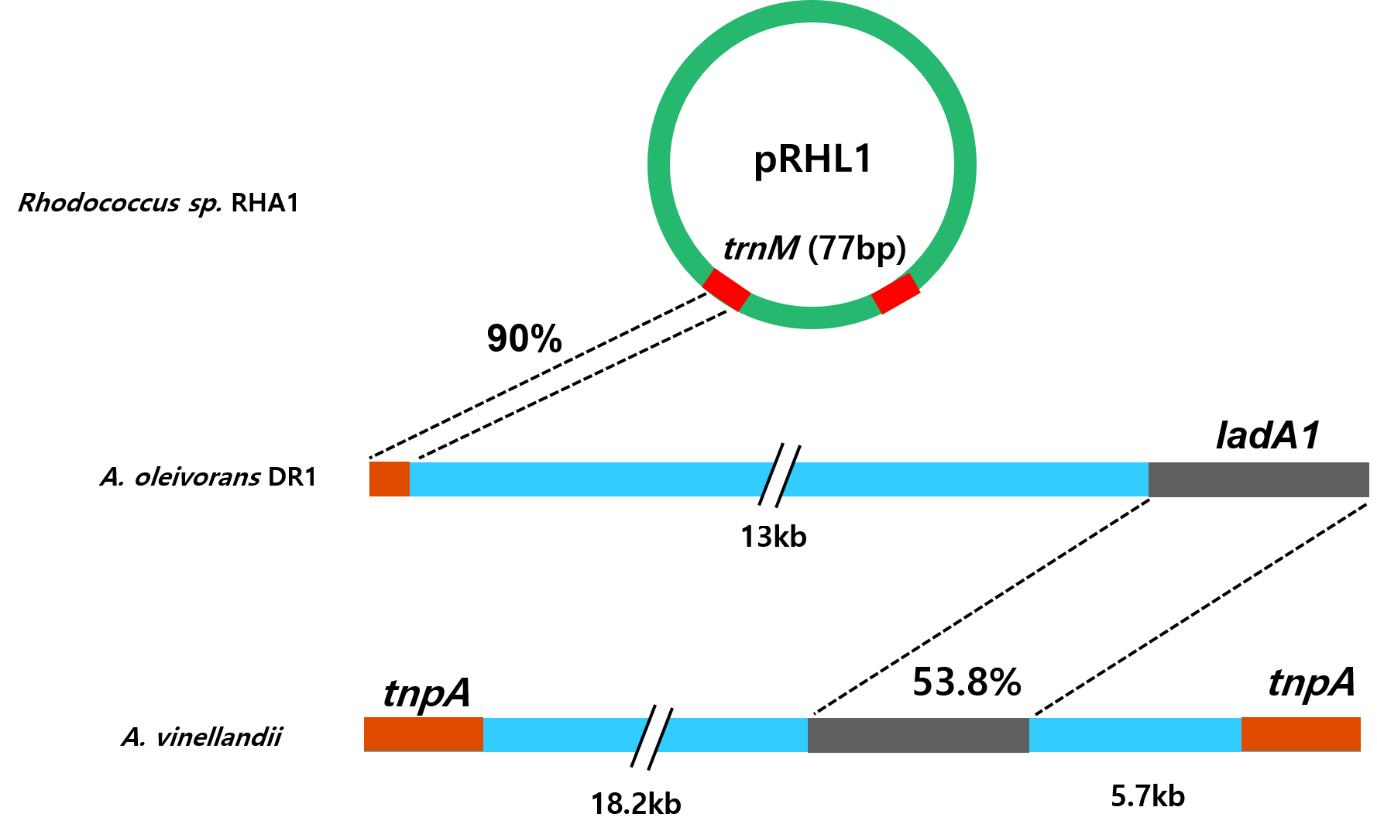


**Fig. S6**

**
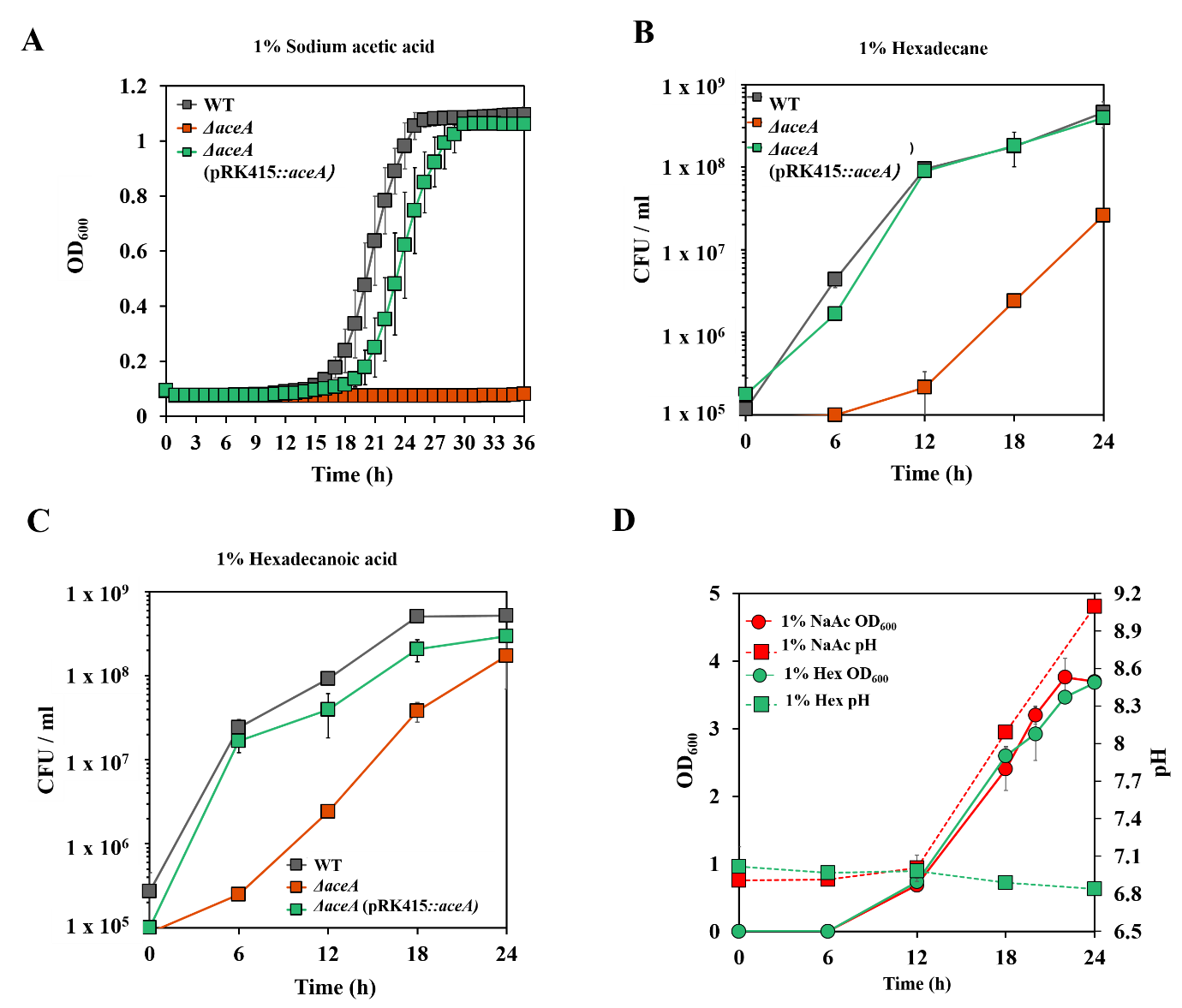
**

**Fig. S7**

**
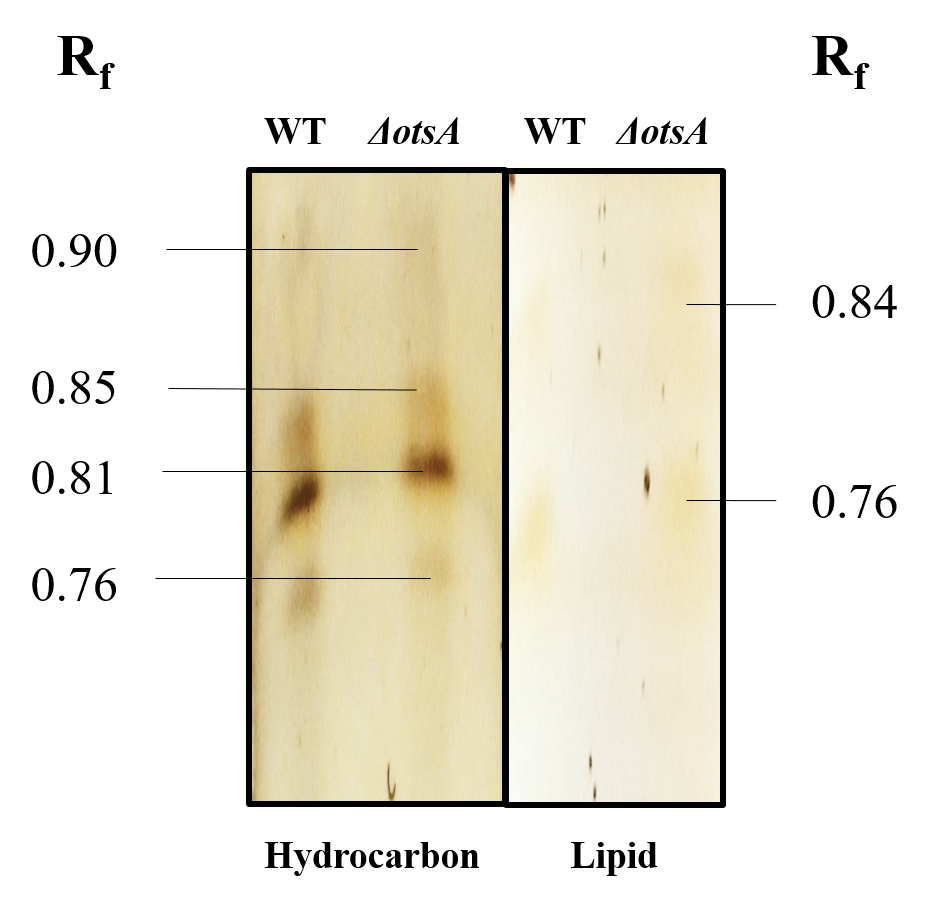
**

**Fig. S8**

**
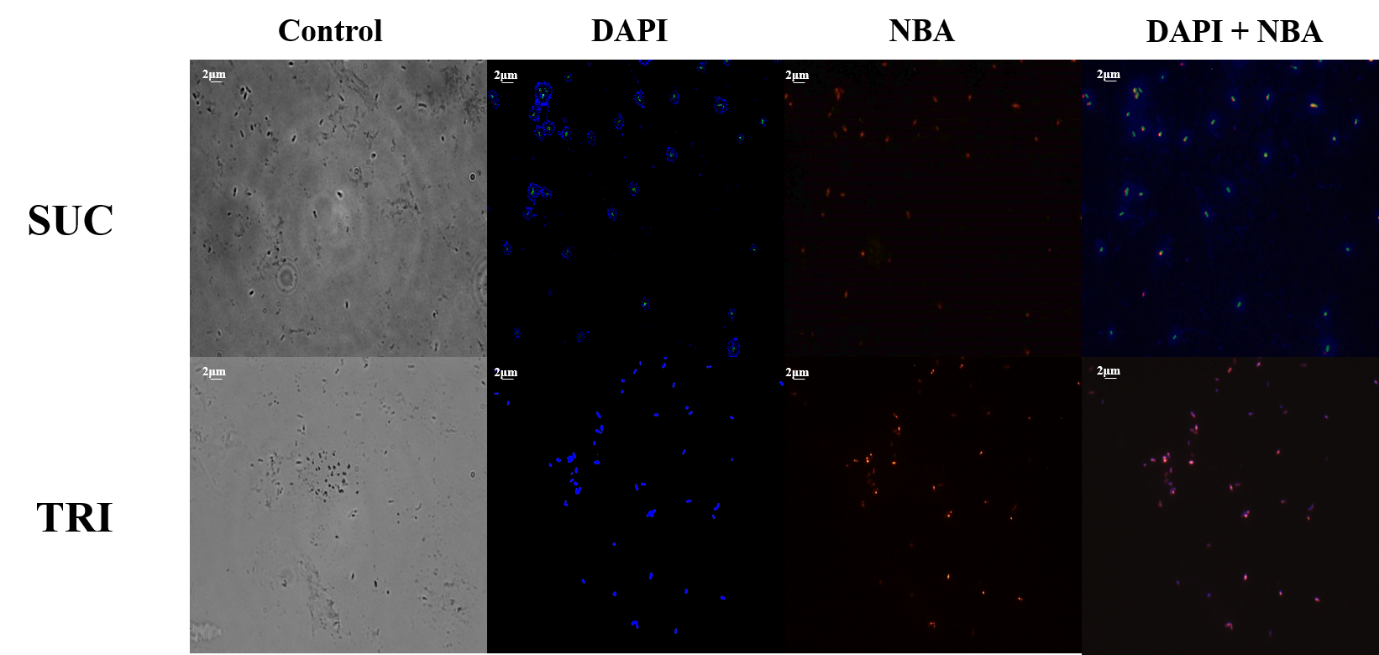
**
